# Differential compartmental processing and phosphorylation of pathogenic human tau and native mouse tau in the line 66 model of frontotemporal dementia

**DOI:** 10.1074/jbc.RA120.014890

**Published:** 2021-01-13

**Authors:** Nora Lemke, Valeria Melis, Dilyara Lauer, Mandy Magbagbeolu, Boris Neumann, Charles R. Harrington, Gernot Riedel, Claude M. Wischik, Franz Theuring, Karima Schwab

**Affiliations:** 1 Charité-Universitätsmedizin Berlin, Berlin, Germany; 2 Bundesanstalt für Materialforschung und-prüfung, Berlin, Germany; 3 School of Medicine, Medical Sciences and Nutrition, University of Aberdeen, Foresterhill, Aberdeen, United Kingdom; 4 Proteome Factory AG, Berlin, Germany; 5 TauRx Therapeutics Ltd., Aberdeen, United Kingdom

**Keywords:** mouse model, Tau protein, protein aggregation, mass spectrometry, frontotemporal dementia, tauopathy, synaptosome

## Abstract

Synapse loss is associated with motor and cognitive decline in multiple neurodegenerative disorders, and the cellular redistribution of tau is related to synaptic impairment in tauopathies, such as Alzheimer's disease and frontotemporal dementia. Here, we examined the cellular distribution of tau protein species in human tau overexpressing line 66 mice, a transgenic mouse model akin to genetic variants of frontotemporal dementia. Line 66 mice express intracellular tau aggregates in multiple brain regions and exhibit sensorimotor and motor learning deficiencies. Using a series of anti-tau antibodies, we observed, histologically, that nonphosphorylated transgenic human tau is enriched in synapses, whereas phosphorylated tau accumulates predominantly in cell bodies and axons. Subcellular fractionation confirmed that human tau is highly enriched in insoluble cytosolic and synaptosomal fractions, whereas endogenous mouse tau is virtually absent from synapses. Cytosolic tau was resistant to solubilization with urea and Triton X-100, indicating the formation of larger tau aggregates. By contrast, synaptic tau was partially soluble after Triton X-100 treatment and most likely represents aggregates of smaller size. MS corroborated that synaptosomal tau is nonphosphorylated. Tau enriched in the synapse of line 66 mice, therefore, appears to be in an oligomeric and nonphosphorylated state, and one that could have a direct impact on cognitive function.

Tau proteins belong to the microtubule-associated protein (MAP) family. They play an important role in the assembly of tubulin monomers into microtubules, which constitute the neuronal microtubule network (1). In the 1970s, the microtubule-associated protein tau was identified as a polymerization factor for microtubules, which promotes microtubule assembly and provides axonal stabilization (2). Fragmented tau proteins constitute the major components of intra-neuronal lesions, paired helical filaments (PHFs) characteristic of Alzheimer's disease (AD) brain (3) and other tau-related neurodegenerative disorders referred to as “tauopathies” (4). The human tau gene (*MAPT)* is encoded on chromosome 17 and alternative splicing of exons 2, 3, and 10 of the primary transcript leads to expression of 6 tau isoforms in the central nervous system (CNS) (5). N-terminal projection domains of tau isoforms contain 0, 1, or 2 inserts, and these determine the spacing between microtubules and the diameter of axons (6, 7). The C-terminal microtubule-binding domains contain either 3 or 4 tandem-repeat domains (R1–R4) that serve as regions that interact with microtubules (8, 9). In addition to their role in promoting microtubule stability and organization, tau may also be involved in the regulation of axonal guided transport through interactions with motor proteins and other binding partners (for review see Ref. 10).

In the longest human CNS tau isoform of 441 amino acid residues, more than 85 putative phosphorylation sites have been reported, most of which are located near the microtubule-binding repeat domains (11, 12). Phosphorylation of tau modulates its affinity to microtubules and multiple kinases and phosphatases regulate tau phosphorylation (10, 13, 14, 15). Hyperphosphorylation inhibits tau–tubulin binding (16, 17) and has been proposed as being important for tauopathies (18, 19). Other factors, such as glycosylation, caspases, and chaperones may also affect both microtubule assembly and tau aggregation (19, 20). Nevertheless, the relevance of tau phosphorylation in disease progression is uncertain. Phosphorylation of tau inhibits tau–tau binding and is preceded by aggregation of nonphosphorylated tau (16, 17) and the aggregation of nonphosphorylated tau is correlated with the onset of cognitive impairment in mice (21).

Irrespective of its phosphorylation state, high affinity binding through the repeat domain (16) and aggregation of truncated tau to form PHFs (22) can be shown to occur *in vitro* in the absence of phosphorylation. Tau aggregation appears to compromise presynaptic release mechanisms, but has little effect on baseline functions of the post-synaptic and hippocampal pyramidal cells (23) and squid axon (24). These data suggest that tau, in addition to its pathogenic role in axons (25, 26), accumulates in dendrites and synapses (27, 28), where it can promote actin polymerization to cross-link with synaptic vesicles, thus restricting their mobilization and lowering neurotransmitter release (29). These data strengthen the view that different tau protein pools may be compartmentalized into various cell spaces, which may be functionally distinct. Both pre- and post-synaptic tau colocalizes with compartment-specific biomarkers in human AD brain (30, 31, 32, 33) indicating that better characterization of the different tau species in these compartments would enable a better understanding of the missorting mechanisms that operate (34, 35). This was examined in our line 66 (L66) mouse as a prototype tauopathy model mimicking frontotemporal dementia (FTD) with parkinsonism overexpressing full-length human tau including mutations P301S and G335D (21).

The focus in this study was on functionally distinct cell compartments, *e.g.* the cytosol and the synapse. In mice, axonal localization of normal tau was confirmed (36, 37), but mislocalization of tau might mediate its pathological propensity (38) with different effects on cell function. Because synapse loss is a good correlate for cognitive capacity in AD (39), we explored how tau localization is affected in these transgenic mice. We report that mutant human tau forms aggregates that do not incorporate endogenous mouse tau and that nonphosphorylated, insoluble human tau protein is enriched in synapses, whereas tau species found in axons and soma are phosphorylated.

## Results

**Figure 1:**
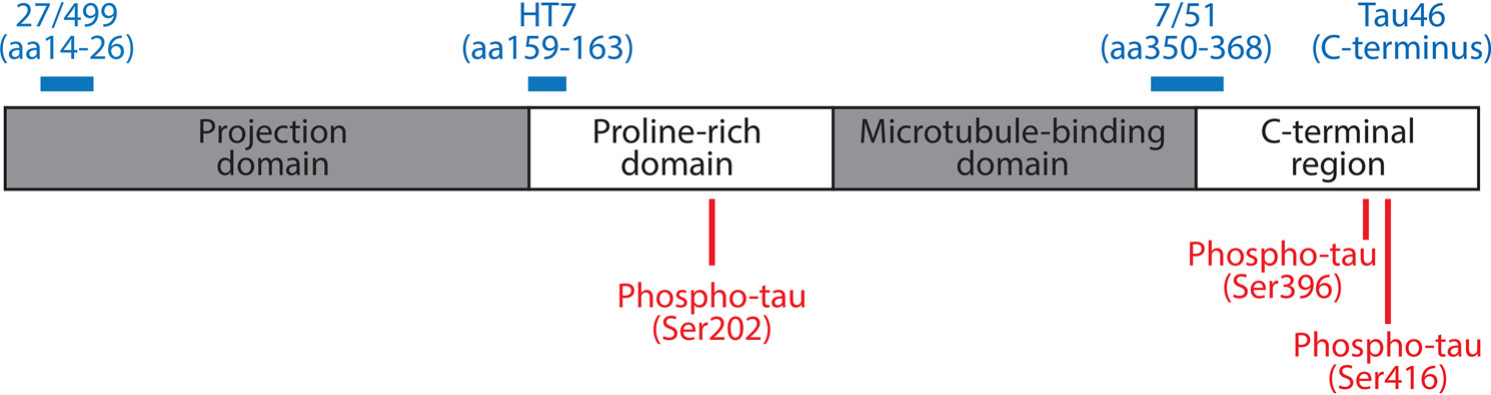
**Schematic representation of epitopes of anti-tau antibodies.** The epitopes recognized by the different anti-tau antibodies used in this work are indicated with numbering based on the longest human CNS tau isoform (2N4R; 441 amino acids). Detailed description of antibodies, including IgG class and supplier are listed in Table 1.

We here characterized the cellular compartmentalization of tau protein species in L66 transgenic mice that express full-length mutant human (htau40 with P301S and G335D mutations) under the control of the *Thy1*-regulatory element with an early onset and widely distributed tau pathology. We applied a range of tau antibodies (Fig. 1 and Table 1) to explore (*a*) whether different aggregation products of the transgenic human tau are compartmentalized, and (*b*) whether the phosphorylation state differs between compartments within the same neurons (*i.e.* soma, cytosol, synapse). Initial immunohistochemical findings were then explored and corroborated through subcellular biochemical fractionation.

**Table 1:** Description of antibodies used in this study, including dilutions used for immunohistochemistry (IHC), immunofluorescence (IF), and immunoblotting (IB)

Antibody^a^	Species/class	Immunogen	Epitope	Supplier (Reference)	Product	Dilution		
						IHC	IF	IB
mAb 7/51	Mouse IgG1κ	PHF-tau	Tau 350-368	TauRx Therapeutics (88)	7/51	1:2,000	NA^b^	1:100
mAb Tau46	Mouse IgG1κ	Bovine tau	Tau carboxyl terminus	Cell Signaling Technology (89)	#4019	1:2,000	NA	1:2,000
mAb 27/499	Mouse IgG2bκ	2N4R tau	Tau 14-26	TauRx Therapeutics (79)	27/499	1:200	NA	NA
mAb HT7	Mouse IgG1κ	Purified human tau	Tau 159-163	Thermo Scientific (90)	MN1000	1:5,000	1:5,000	1:2,000
pAb pSer-202-tau	Rabbit IgG	Synthetic peptide corresponding to residues surrounding Ser-202 of human tau protein	Tau pSer-202	Cell Signaling (91)	#11834	1:500	1:500.	NA
mAb pSer-396-tau	Mouse IgG2b	Purified human tau	Tau pSer-396	Cell Signaling (92)	#9632	1:2,000	1:2,000	NA
mAb pSer-416-tau	Rabbit IgG	Synthetic phosphopeptide corresponding to residues surrounding Ser-416 of human Tau protein	Tau pSer-416	Cell Signaling (93)	#15013	1:500	1:500	NA
pAb β-actin (C-11)	Goat IgG	β-actin	Actin carboxyl-terminus	Santa Cruz Biotechnology	sc-1615	NA	NA	1:1,000
mAb synapsin-1	Mouse IgG1κ	Synapsin-1a and 1b	Synapsin 1 proline rich D domain	Synaptic Systems (94)	106001	NA	1:2,000	1:10,000
mAb synapsin-2	Rabbit IgG	hSynapsin-2	Synapsin-2 Gly-503	Cell Signaling	#85852	NA	1:2,000	NA
mAb SNAP25	Mouse IgG1	Crude synaptic preparation from the human post-mortem brain	NA	Biolegend (95)	#MMS-614P	NA	NA	1:1,000
mAb syntaxin	Mouse IgG1	Crude synaptic preparation from the human post-mortem brain	Syntaxin 4-190	Abcam	ab112198	NA	NA	1:20,000
mAb PSD95	Rabbit IgG	hPSD95	PSD95 Gln-53	Cell Signaling (96)	#3450	NA	NA	1:500


a mAb, monoclonal antibody; pAb, polyclonal antibody.

b NA, not applicable for this study.

### Histopathological confirmation of hTau in all neuronal compartments

**Figure 2:**
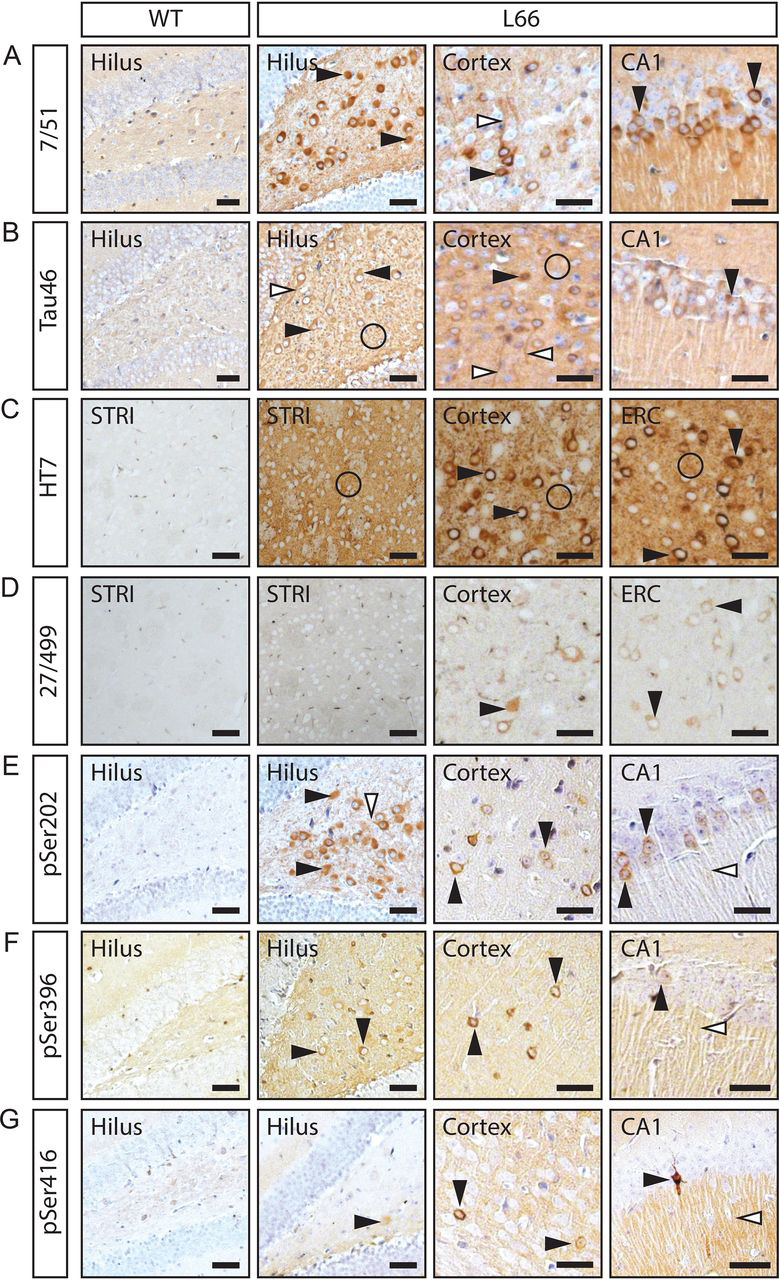
**Tau immunohistochemistry in L66 and WT control mice.** Tau immunoreactivity with phosphorylation-independent antibodies 7/51 (*A*), Tau46 (*B*), HT7 (*C*), and 27/499 (*D*), and with phosphorylation-specific antibodies pSer-202 (*E*), pSer-396 (*F*), and pSer-416 (*G*) show prominent intraneuronal staining in cortex and hippocampus in L66 brains. Phosphorylation-independent antibodies showed strong synaptic staining mainly in striatum in L66 (*A–D*). Phosphorylation-specific antibodies showed strong staining in cell bodies and axons in L66 (*E–G*). *Black arrowhead,* cytosolic staining in neurons; *white arrowhead*, axonal/dendritic staining; *circle*, synaptic staining. *CA1*, hippocampus subfield CA1; *STRI,* striatum. *Scale bars*, 100 μm. *L66*, line 66 tau transgenic mice.

We first established histologically that transgenic human tau accumulates in cell bodies, dendrites, and synapses in L66 mice (Fig. 2 and Fig. S1). Immunopositive labeling was confirmed for all antibodies in cortex, striatum, and hippocampus of L66, whereas sections from WT controls were devoid of tau immunoreactivity with the panel of antibodies we have used spanning a number of domains across the tau molecule (Fig. 1). Labeling with phosphorylation-independent tau antibodies 7/51 (Fig. 2*A*), Tau46 (Fig. 2*B*), and HT7 (Fig. 2*C*) was observed in the somata of pyramidal cells in CA1, hilus of the dentate gyrus, ERC (entorhinal cortex) and cortex (see *black arrowheads*, Fig. 2), but also in cortical and hippocampal dendrites (Fig. 2, *white arrowheads*) and in nerve terminals in cortex and striatum (Fig. 2*C*, *circles*). Human-specific N-terminal tau mAb 27/499 (Fig. 2*D*) showed a similar pattern of labeling. The pattern of labeling seen with phosphorylation-dependent antibodies was distinct. Immunoreactivity for pSer-202 (Fig. 2*E*), pSer-396 (Fig. 2*F*), and pSer-416 (Fig. 2*G*) were seen mainly in cell bodies and axons of principal cells in cortex, the hilus, and CA1, but not in nerve terminals.

**Figure 3:**
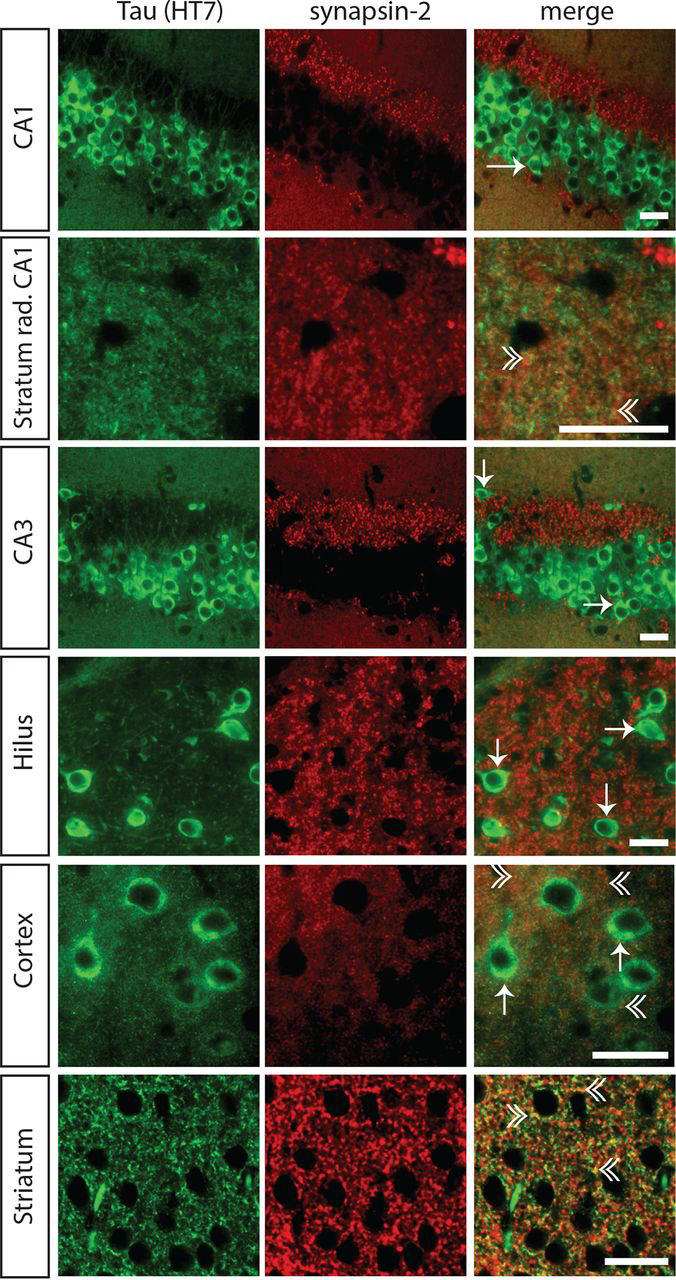
**Synaptic localization of tau in L66 using immunofluorescence.** Tau immunoreactivity with the phosphorylation-independent antibody HT7 is localized in the neuronal soma (*arrows*), dendrites, and axons of neurons in hippocampal CA1 and CA3, cortex and hilus of the dentate gyrus. Co-localization of HT7-reactive tau and the synaptic marker synapsin-2, seen as yellow puncta in the merged images (*double arrowhead*), confirms the synaptic accumulation of transgenic tau. Synaptic staining of tau is evident mainly in striatum and, to a lesser extent, in cortex and stratum radiatum of the CA1. *Green,* tau; *red,* synapsin-2. *Arrow,* staining in the neuronal soma; *double arrowhead*, synaptic staining. *Scale bars*, 25 μm.

Tau accumulation in synapses was verified by double labeling of sections with HT7 (mouse) and the synaptic protein synapsin-2 (rabbit). Because synapsin-1 (used for biochemical assays, see below) and synapsin-2 are distributed similarly within the pre-synapse (40), synpasin-2 was used for double labeling as it was the only synapsin antibody available not raised in mouse. Tau recognized by HT7 was found to be co-localized with synapsin-2, confirming its synaptic localization (Fig. 3). Synaptic tau was seen mainly in striatum, cortex, and stratum radiatum of CA1, but was absent in other hippocampal regions. Phospho-tau was virtually absent from synapses (Fig. S2). These data confirm that transgenic mutant human tau is present in all compartments of the neuron with some region-specific variation in labeling in synaptic endings.

### Subcellular fractionation confirms hTau in all compartments of L66 neurons

**Figure 4:**
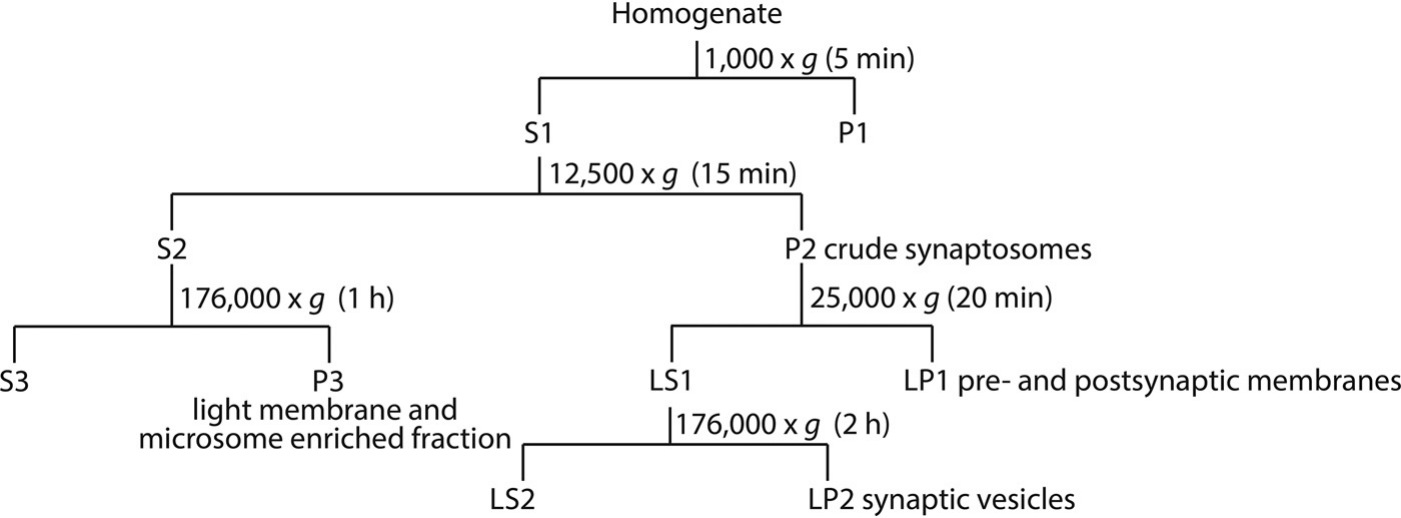
**Extraction protocol for subcellular fractionation.** Brain tissue homogenate was centrifuged at speeds as indicated in the diagram to allow separation of synaptic from cellular fractions.

To characterize better the compartmental localization of human tau in L66 mice, we next applied ultracentrifugation and subcellular fractionation of brain extracts. The procedure for generating the fractions is depicted in Fig. 4. Importantly, P1 and P3 are pellets derived from the soma of cells, whereas P2 is a crude synaptosomal fraction. Further centrifugations of P2 produced LP1 (a mix of pre- and post-synaptic compartments) and LP2 as the enriched synaptic fraction. The cytosol is represented by fractions S1–S3. The LS1 fraction was used as a reference for quantitative comparisons as it had the lowest levels of the proteins of interest (relative protein level (RPL) equals 1).

**Figure 5:**
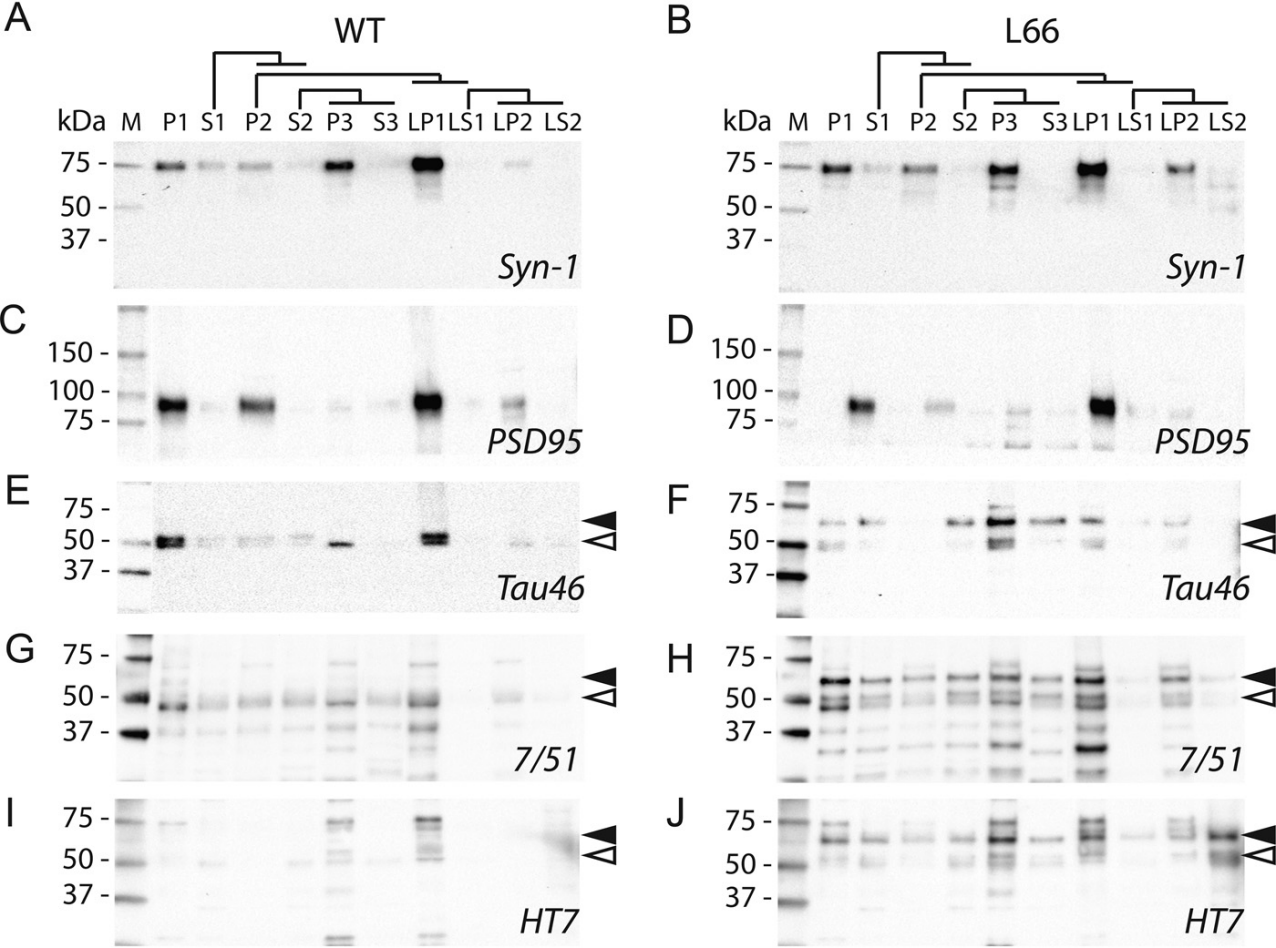
**Cellular distribution of tau using subcellular fractionation.** Brain tissue from WT controls (*left panel*) and L66 (*right panel*) were subjected to subcellular fractionation as described under “Experimental procedures.” Sample fractions (1 µg of total protein) were separated on 4–20% gradient glycine-SDS-PAGE and representative immunoblots are shown. Antibodies against synapsin-1 and PSD95 (*A–D*) were used to confirm synaptic enrichment. Antibodies against tau (*E* and *F* for Tau46, *G* and *H* for 7/51, and *I* and *J* for HT7) were used to quantify tau in the different fractions. Transgenic tau, reactive with 7/51 and HT7 was evident in most fractions of L66 mice, visible as a 62-kDa band (*black arrowheads*), whereas no specific signal for WT mice was seen using either antibody. A tau band at around 50-55 kDa (*white arrowheads*), reactive with the antibody Tau46, showed a similar enrichment pattern for endogenous tau, both in WT controls (*E*) and L66 (*F*). *Black arrowheads*, 62-kDa band, specific to L66 tau-transgenic mice. *White arrowheads*, 50-55-kDa band, murine tau. *L66,* line 66 tau transgenic mice. All 10 bands corresponding to transgenic tau from different fractions were excised from silver-stained glycine gels for downstream MS analyses and results are shown in Table 2 and Tables S1 and S2.

**Figure 6:**
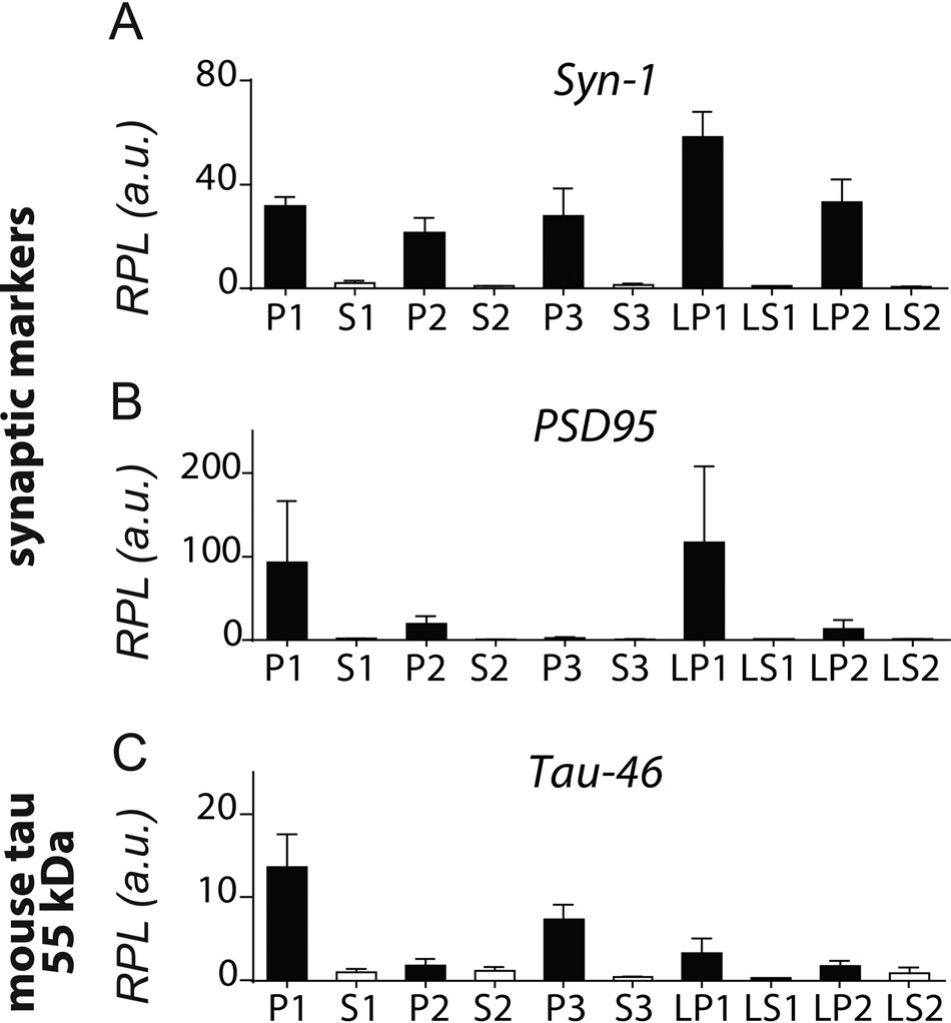
**Quantification of synaptic markers and tau after subcellular fractionation in WT mice.** Densitometric quantification of synapsin-1 (*A*), PSD95 (*B*), and Tau-46 reactive mouse tau at 50-55 kDa (*C*) was conducted with Image Laboratory using stain-free total protein loading for normalization. Data are expressed as mean ± S.E.

**Figure 7:**
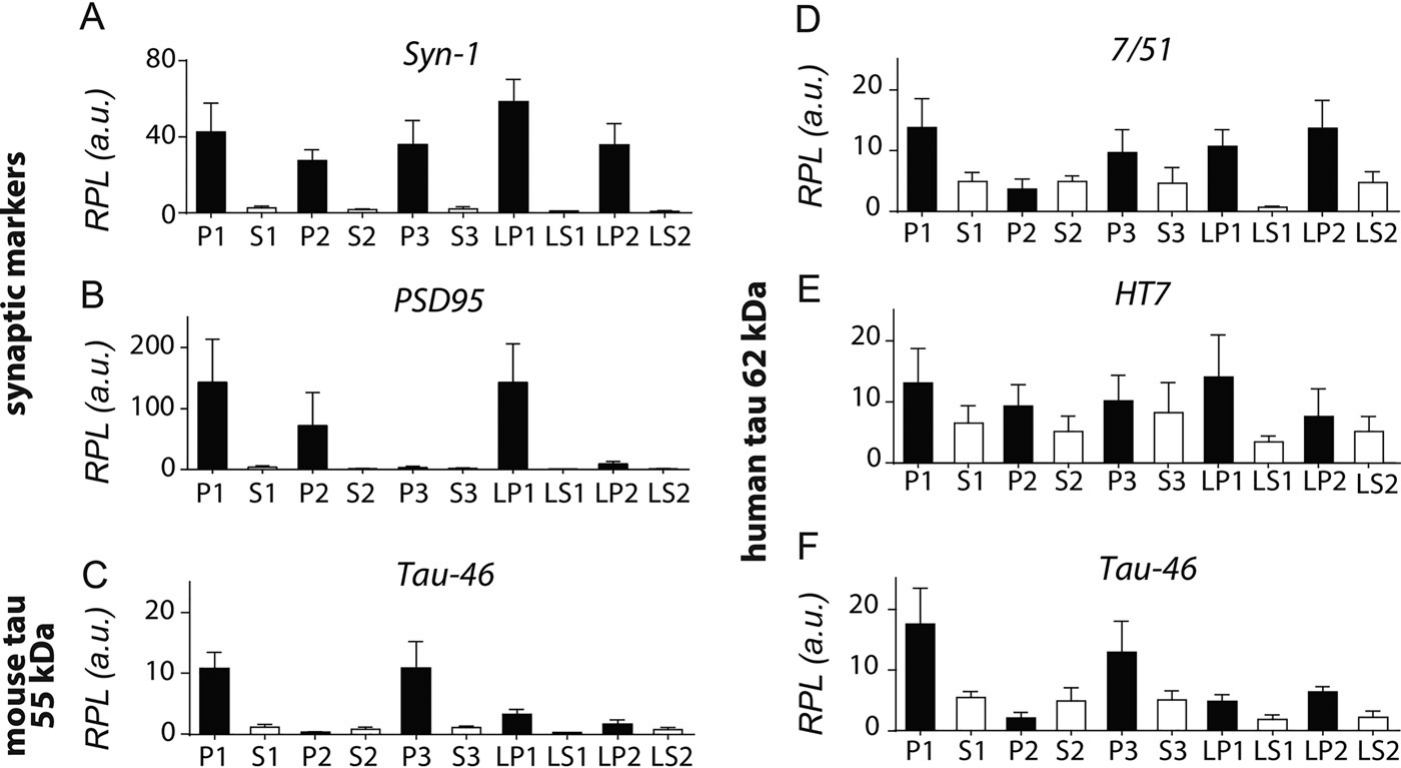
**Quantification of synaptic markers and tau after subcellular fractionation in L66 mice.** Densitometric quantification of synapsin-1 (*A*), PSD95 (*B*), Tau-46 reactive mouse tau at 50-55 kDa (*C*), as well as transgenic human tau at 62 kDa reactive with 7/51 (*D*), HT7 (*E*), and Tau-46 (*F*) was conducted with Image Laboratory using stain-free total protein loading for normalization. Data are expressed as mean ± S.E.

Synaptic enrichment in fractions LP1 and LP2 was confirmed by enrichment of synapsin-1 and PSD95 proteins (Fig. 5, *A*–*D*). As expected, synapsin-1 was highly enriched in all pellet fractions (Fig. 5, *A* and *B*; for quantification see Figs. 6*A* and 7*A*) in both WT controls and L66 tissue. Consistent with a previous report (41), the most highly purified synaptic vesicle fraction LP2 and the pre- and postsynaptic membrane fraction LP1 had 40- to 60-fold enrichment of synapsin-1 compared with LS1. PSD95 was found mainly in pellets P1, P2, and in the pre- and postsynaptic membrane fraction LP1 (Fig. 5, *C* and *D*, and Figs. 6*B* and 7*B* for quantification) as reported by others (34, 42).

Murine tau was quantified using mAb Tau46 (Fig. 5, *E* and *F*, *white arrowheads*). Although this antibody labels tau in both WT and L66 brain preparations, murine tau has a gel mobility of 50-55 kDa. There was a 10- to 15-fold enrichment of murine tau in the crude pellet fractions P1 and P3 (quantification in Figs. 6*C* and 7*C*). There was relatively little enrichment of the 50-55–kDa murine tau in LP1 (3.5-fold) and LP2 (2-fold).

Transgenic mutant tau has a distinct gel mobility of 62 kDa, which could be detected using phosphorylation-independent antibodies 7/51, HT7, and Tau46 (Fig. 5, *E*–*J*, *black arrowheads*) and this was seen only in brain tissues from L66 mice and not in WT samples. Transgenic 62-kDa tau was detected in all pellet fractions with antibodies 7/51 (Fig. 7*D*, 10- to 15-fold), as well as with HT7 and Tau46 (Fig. 7, *E* and *F,* 3- to 6-fold). Of particular interest, the co-purification of the 62-kDa human tau with synaptic proteins in the synaptic LP1 and LP2 fractions corroborates the co-localization established by immunohistochemistry (Fig. 3).

### Synaptic hTau in L66 is nonphosphorylated

We further explored the phosphorylation status of human and murine tau using MS. This approach was necessary given the large number of phosphorylation sites of tau that have been reported and the limited amounts of tau protein available from LP1 and LP2. Application of MS has the further advantage that it can detect the presence of multiple phosphorylation sites in a single sample run. The 62-kDa tau bands specific to L66 found in the various subcellular extracts (Fig. 5*J*) were excised from silver-stained gels and subjected to Orbitrap LC–MS. Bands were taken in duplicates and digested with either trypsin or thermolysin to increase sequence coverage.

Transgenic mutant human tau was found in all nine fractions (Table 2 and Table S1 and Figs. S3–S7 for detailed MS data) corroborating the results from immunoblotting (Fig. 5). With the PEAKS software, we established phosphorylation of transgenic tau at Thr-181, Ser-199, Ser-202, and Thr-231 (Table 2 and details in Table S2 and Figs. S3–S7) in fractions S1, S2, S3, and LP1. Of particular interest, no phosphorylated residues were identified in fractions LS1, LS2, and LP2. Because these represent the subfractions derived from the crude P2 synaptosomal preparation, this implies that the transgenic human tau found in synapses was not phosphorylated in any of the peptide fragments that were analyzed.

**Table 2:** Subcellular fractions (see Fig. 4 for fractions) probed by Orbitrap LC-MS and identified by Mascot and PEAKS search engines Phosphorylated tau peptides were identified by PEAKS. Only the transgenic human tau isoform Tau-X is shown. For detailed MS results, see Tables S1 and S2.

Subcellular Fraction	Number of peptides matched (Mascot/PEAKS)	Number of phosphorylated peptides	Phosphorylation sites
S1	18/21	4	Thr-181, Ser-202, Thr-231
P2	4/5	None	-
S2	17/28	6	Thr-181, Ser-199, Ser-202, Thr-231
P3	14/14	None	-
S3	16/22	4	Thr-181, Thr-231
LP1	13/20	4	Thr-181, Ser-202, Thr-231
LS1	6/6	None	-
LP2	3/2	None	-
LS2	1/1	None	-

### Urea resistance of large hTau aggregates

**Figure 8:**
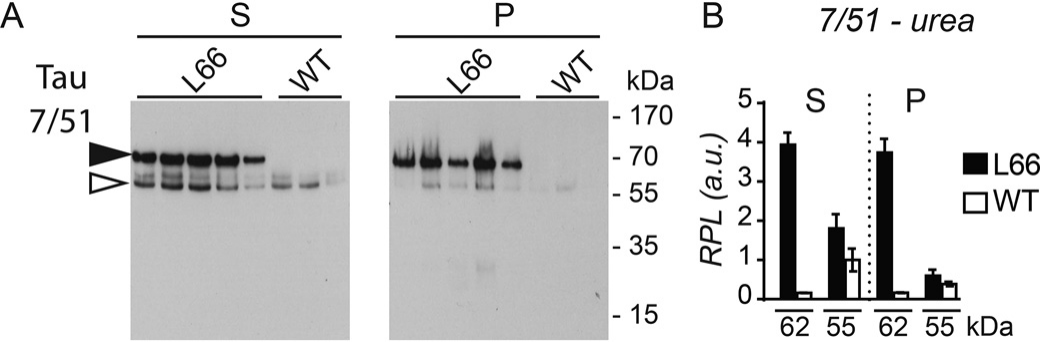
**Urea solubility of tau species.** Brain tissue from L66 and WT control mice (*n* = 5 and 3, respectively) was subjected to urea protein extraction and supernatant (*S*) and soluble pellet (*P*) fractions were obtained by low-speed centrifugation (16,000 × *g* for 45 min). Protein extracts (20 µg) were separated on 10% Tricine-SDS-PAGE and the immunoreactivity of the fractions was probed using mAb 7/51 as shown in immunoblots (*A*). Densitometric quantification of the blots revealed 7/51-reactive tau specific to L66 (62-kDa bands, *black arrowhead*) accumulated in P and S nearly to the same extent, emphasizing that some of the transgenic tau was resistant to urea solubilization (*B*). Murine tau detected at 55 kDa (*white arrowhead*) was almost completely depleted from the pellet P after addition of urea. Densitometric quantification was conducted with AlphaEase using the 55 kDa band in S from WT for normalization. Values are expressed as mean ± S.E.

Given that transgenic mutant human tau differs from native murine tau in terms of compartment distribution and phosphorylation status, we sought to understand better the differences in solubility in 7 m urea, which disrupts membrane interactions and hydrogen bonding (Fig. 8). Tau with 62-kDa gel mobility detected with mAb 7/51 was found in approximately equivalent amounts in the urea supernatant and pellet fractions after low-speed centrifugation (16,000 × *g* for 45 min). The levels in L66 mice were 4-fold higher in both fractions relative to the 55-kDa murine tau released into the urea supernatant (Fig. 8*A, black* and *white arrowheads,* respectively, and Fig. 8*B* for quantification). By contrast, murine tau was found at a lower level in the urea supernatant from WT mice compared with L66 mice and was largely absent from urea pellet in WT mice (Fig. 8*B*, 1 to 2-fold).

### Mutant hTau is partially insoluble in Triton X-100

**Figure 9:**
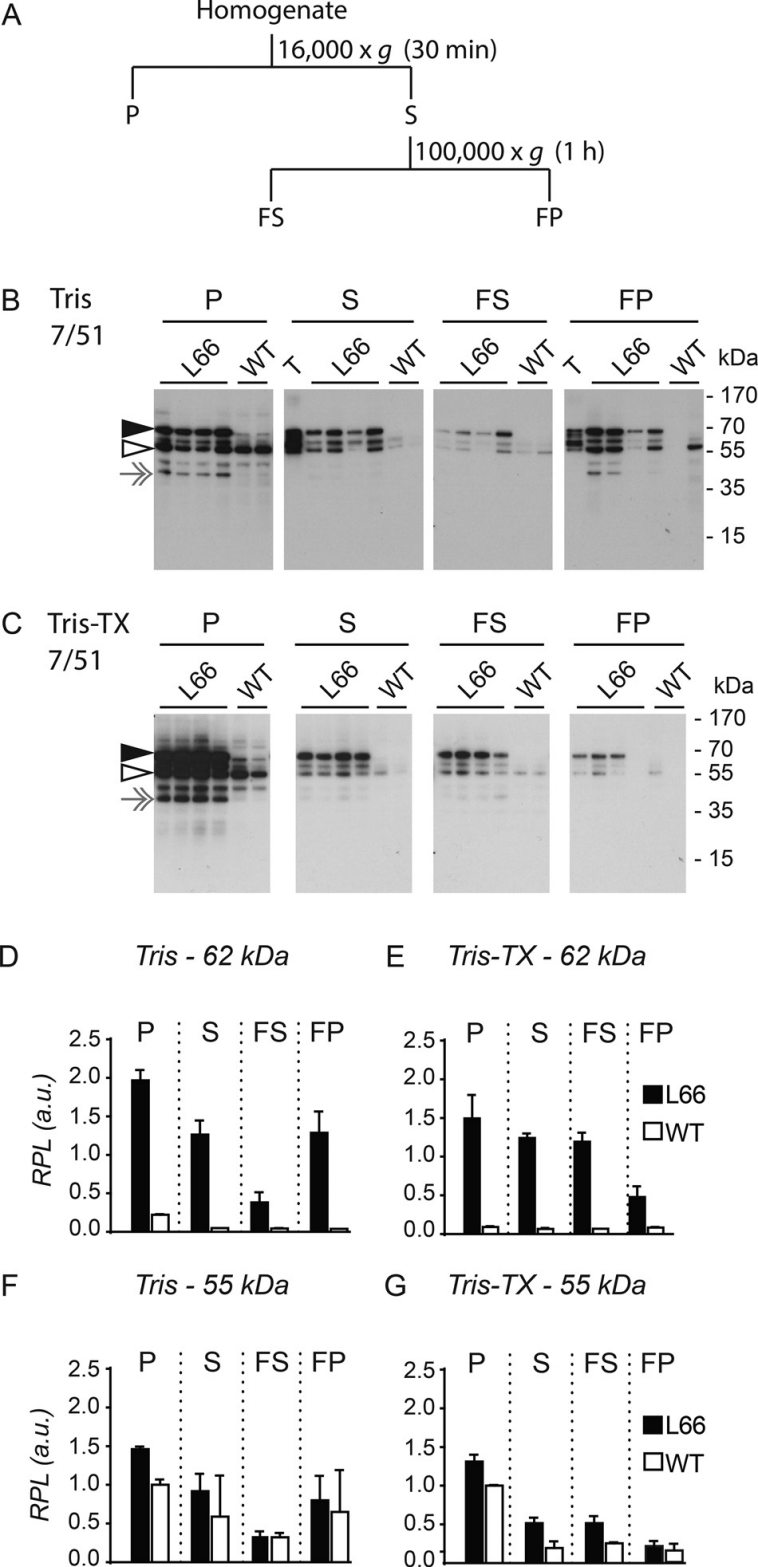
**Tau protein solubility with and without Triton X-100.** (*A*) Ex-traction scheme for low- and high-speed extraction of brain tissue from L66 tau transgenic and WT control mice; tissue was subjected to extraction with either Tris (*B*) or Tris-Triton X-100 (*C*). Pellet (*P*) and supernatant (*S*) fractions were obtained by low-speed centrifugation at 16,000 × *g* for 30 min, whereas the fast pellet (*FP*) and the fast supernatant (*FS*) fractions were obtained by high-speed centrifugation of S at 100,000 × *g* for 1 h. Samples (15 µg of protein) were separated by 10% tricine-SDS-PAGE and immunoblots are shown. The immunoreactivity of the fractions was probed using the phosphorylation-independent anti-tau antibody 7/51. Densitometric quantification of the blots revealed 7/51-reactive tau (62-kDa bands) accumulated in P, S, and FP fractions. After the addition of Triton X-100, some of 7/51-reactive tau in FP fraction was solubilized and found in the FS fraction. Densitometric quantification was conducted with AlphaEase using the 55-kDa band in P from WT for normalization. Values are expressed as mean ± S.E. *T*, tau ladder. *Black arrowheads*, 62-kDa human tau, and *white arrowheads*, 55-kDa mouse tau. *Gray arrows*, 37-kDa band, non-tau nonspecific labeling.

We used differential centrifugation in the presence or absence of Triton X-100 to compare the distribution of mutant human tau and native murine tau using mAb 7/51 in immunoblots (Fig. 9). As noted above, the 62-kDa tau species was absent in specimens from WT mouse brain, whereas murine tau species with gel mobility in the 50–55 kDa range were present in both L66 and WT specimens. The amounts of 55-kDa tau in the low-speed pellet and supernatant fractions were comparable. Following high-speed centrifugation of the low-speed supernatant, the amounts of 55-kDa tau were again comparable in the high-speed supernatant (FS) and pellet (FP) fractions prepared from L66 and WT mice, with the levels in the pellet approximately double those in the supernatant (Fig. 9*F*). When the same procedure was carried out in the presence of Triton X-100 the amounts in the pellet from L66 and WT mice were both substantially reduced in the high-speed pellet (Fig. 9*G*).

Compared with the level of 55-kDa tau in the low-speed pellet, there was 2-fold enrichment of 62-kDa tau in the same pellet and 1.3-fold enrichment in the corresponding supernatant from L66 mice (Fig. 9*D*). Following high-speed centrifugation of the supernatant, the majority of the 62-kDa tau was in the pellet (1.3-fold enrichment) with little remaining in the supernatant (0.4-fold RPL). This distribution was reversed when the same procedure was carried out in the presence of Triton X-100, with the majority now in the high-speed supernatant (1.2-fold), but with a 0.5-fold RPL remaining in the pellet (Fig. 9*E*). Therefore, approximately half of the 62-kDa tau from L66 mice is resistant to solubilization by Triton X-100. Because Triton X-100 would be expected to solubilize any membrane complexes, we interpret this as indicating that the 62-kDa tau sedimenting in these conditions is in the form of oligomeric complexes of molecular mass of at least 500 kDa. Similar findings were seen using HT7 (data not shown).

### Soluble hTau is stable to heat denaturation

**Figure 10:**
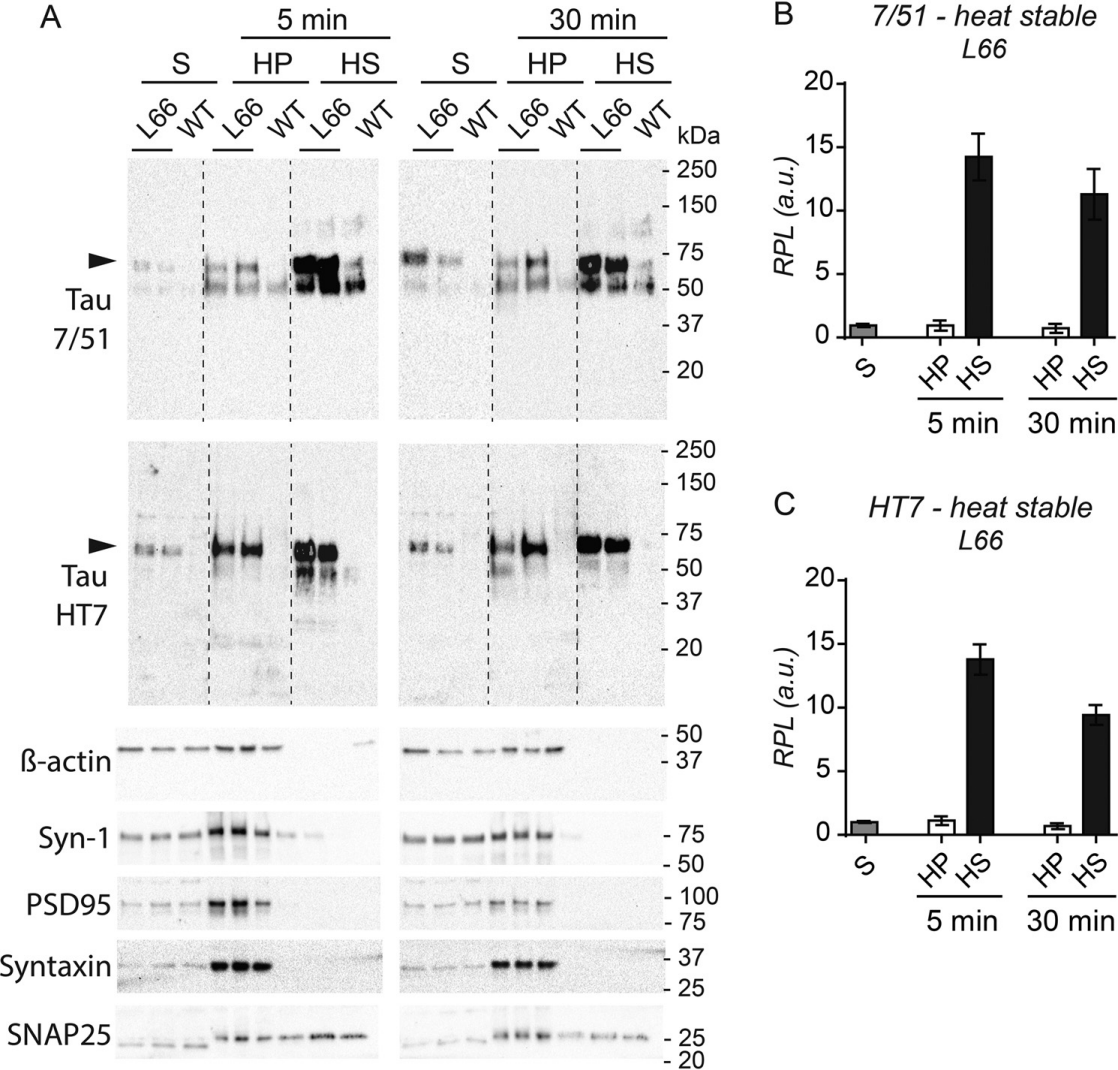
**Heat stability of Tris-soluble tau.** Tris-soluble fractions (see Fig. 9, S fractions) were heated at 95 °C for 5 or 30 min and 2 µg of total protein of original supernatant (S), the heat-stable supernatant (HS), and precipitated pellet fractions (HP) were subjected to 4–15% glycine SDS-PAGE and subsequent immunoblotting using the phosphorylation-independent anti-tau antibodies 7/51 and HT7 as shown in representative immunoblots (*A*). With both antibodies, heat-treated tau was mainly found in the supernatant fraction HS, and only a small subset of tau was found in the pellet HP. β-Actin and several synaptic proteins were solely found in HP after only 5 min heat treatment. *Dotted lines* indicate where fractions from one brain were excised due to sample smearing. The quantification in *panels B* and *C* represent data obtained from (*A*) and from further samples. Synapsin-1, PSD95, and syntaxin were not heat stable, whereas SNAP25 was partially stable toward heat treatment, as some of it remained soluble in the supernatant HS after 5 min of boiling. Densitometric quantification for 7/51 (*B*) and HT7 (*C*) conducted with Image Laboratory using stain-free total protein loading for normalization confirmed the findings for transgenic tau, emphasizing the thermal stability of tau. Values are expressed as mean ± S.E. *Black arrowheads*, 62-kDa human tau.

We further compared the heat stability of mutant human and native murine tau species isolated from the low-speed supernatant in brain extracts from L66 and WT mice. As seen in Fig. 10, there is substantial selective enrichment of the 62-kDa tau species in the supernatant following heat treatment for 5 or 30 min at 95˚C. There is minimal corresponding enrichment of native murine tau. The results were similar for both phosphorylation-independent antibodies 7/51 and HT7 (Fig. 10*A*, *black arrowheads*). The enrichment of human tau was more than 10-fold compared with that present in the low-speed supernatant (Fig. 10, *B* and *C*). By contrast, β-actin, synapsin-1, PSD95, and syntaxin were completely absent from the heat-stable supernatant (HS) after only 5 min of heat treatment (Fig. 10*A*); the same was the case for synaptophysin and synapsin-2 (data not shown), whereas SNAP25 showed some resistance toward treatment with heat. Therefore, unlike transgenic tau, other membrane-interacting and synaptic proteins are not resistant to heat denaturation.

## Discussion

L66 mice overexpress the longest human CNS tau isoform of 441 amino acid residues, which contains the aggregation-promoting mutations P301S and G335D. Expression of mutant human tau is widespread in the brain in this model producing prominent tau aggregates in neurons that can be labeled with Bielschowsky silver and primulin (21). We have now extended the studies in L66 with the aim of understanding better the differential compartmental localization of full-length native mouse and transgenic mutant human tau and how the phosphorylation status of mutant tau varies according to its compartmental localization within neurons. The key novel findings that we report are that although mutant human tau appears to have a wide distribution by immunohistochemistry, biochemical analysis reveals that its compartment distribution differs in a number of respects from that of native tau. Whereas native tau is largely soluble in urea and detergent, a substantial proportion of mutant tau in the brain is insoluble in both. Of particular interest, we report that mutant tau is co-localized with synaptic markers histologically and co-purifies with synaptic proteins in synaptosomal and synaptic vesicle preparations. Immunoblotting and sequence analysis of peptide digests of synaptic mutant tau reveal that it is largely unphosphorylated, whereas it is phosphorylated in cell body and axons (summarized in Table 3). Therefore, the differential neuronal processing of mutant human tau relative to native murine tau, particularly in synapses, may underlie its adverse effects on behavior.

**Table 3:** Summary for subcellular distribution of tau and its phosphorylation status using mmunohistochemistry, immunofluorescence, subcellular fractionation and MS (see Figs. 2Figure 3, Figure 4, Figure 5^6^ and Table 2) The following designations were used: S1-S3, LS1, LS2: supernatants; P1, nuclei and debris; P2, crude synaptosomes; P3, light membrane/microsomes; LP1, pre- and postsynaptic membranes, LP2, synaptic vesicles.

Method	Protein	Antibody	Genotype	Cellular compartment/fraction									
				Cell body						Puncta			
Immunohistochemistry	Tau	7/51	L66	+					2+				
			WT	−					+				
		HT7	L66	+					3+				
									(co-localization with synapsin-2)				
			WT	−					−				
		Tau46	L66	+					2+				
			WT	−					+				
		27/499	L66	+					−				
			WT	−					−				
		pSer-202	L66	+					+				
			WT	−					−				
		pSer-396	L66	+					+				
			WT	−					+				
		pSer-416	L66	+					+				
			WT	−					+				
				**S1**	**P1**	**S2**	**S3**	**P3**	**P2**	**LS1**	**LP1**	**LS2**	**LP2**
Subcellular fractionation	Synaptic markers	Syn1	L66	−	2+	−	−	2+	2+	−	2+	−	2+
			WT	−	2+	−	−	2+	2+	−	2+	−	2+
		PSD95	L66	−	2+	−	−	−	2+	−	2+	−	−
			WT	−	2+	−	−	−	2+	−	2+	−	−
	Human tau	7/51	L66	2+	2+	2+	2+	2+	2+	+	2+	2+	2+
	(62 kDa)		WT	−	−	−	−	−	−	−	−	−	−
		HT7	L66	2+	2+	2+	2+	2+	2+	+	2+	2+	2+
			WT	−	−	−	−	−	−	−	−	−	−
		Tau46	L66	2+	2+	2+	2+	2+	+	+	2+	+	2+
			WT	−	−	−	−	−	−	−	−	−	−
	Mouse tau	Tau46	L66	−	2+	−	−	2+	−	−	+	−	+
	(55 kDa)		WT	−	2+	−	−	2+	+	−	+	−	+
MS	Phosphorylated human tau	Silver gel (62 kDa)	L66	2+	−	2+	2+	−	−	−	2+	−	−

Two basic features have made it possible to differentiate native mouse tau from mutant human tau in the brain. At the histological level, there is no labeling of native tau in WT mouse brain with any of the monoclonal antibodies we have used spanning N-terminal, repeat domain and C-terminal segments of the molecule under the conditions we have used. This is a well-recognized immunohistochemical feature of native tau using conventional tissue processing with formalin fixation (43). By contrast, transgenic mutant tau can be labeled by all the antibodies we have tested, including both phosphorylation-dependent and phosphorylation-independent monoclonal antibodies. Although the reasons for this difference are not yet fully understood at the molecular level, they indicate that the endogenous processing and consequent epitope exposure of mutant tau is fundamentally different from that of native tau. The biochemical characterization of these differences is made possible by the difference in gel mobility between native mouse and transgenic human tau in immunoblots. Most forms of tau protein are well-known to have atypical gel mobility relative to their molecular weight. This has been shown to be due to altered affinity for SDS, which has the effect of retarding their electrophoretic mobility in gels (5, 44). In particular, mouse tau has gel mobility corresponding approximately to 50-55 kDa, whereas mutant human tau has gel mobility corresponding to ∼62 kDa. This makes it possible to distinguish the two forms of tau in immunoblots despite using monoclonal antibodies, which are not inherently discriminatory.

We first show that mutant human tau co-localizes immunohistochemically with synapsin-2. This was seen mainly in striatum, and to a lesser extent in cortex and stratum radiatum of CA1. We then used biochemical fractionation to determine whether mutant tau co-purifies with pre- and post-synaptic markers (synapsin-1 and PSD95, respectively). Synaptic proteins were found in crude fractions pelleting with sedimentation limits corresponding to a particle of size 95 nm (crude synaptosomal fraction P2 and the LP1 fraction containing pre- and post-synaptic membranes). These were further enriched in the preparations with particle sedimentation limits corresponding to 21 nm (microsomal fraction P3) and 15 nm (synaptic vesicle fraction LP2). Mutant tau protein recognized by the repeat-domain marker (mAb 7/51; residues 350-368) was found to be enriched in the smallest particle fractions P3 (21 nm) and LP2 (15 nm). The same was true to a somewhat lesser extent using more N-terminal (HT7; residues 159-163) and C-terminal (Tau46; residues 404-441) antibodies. Sequencing of peptides from the 62-kDa tau protein co-purifying with synapsin-1 in these fractions identified tau fragments spanning residues 195 to 369 by MS sequence analysis. Although small amounts of native mouse tau were also identified in these fractions, these were present at levels ∼20% of the levels of mutant human tau. Therefore, we have established that soluble tau species from mutant human tau co-localize with synapses and co-purify with synaptic proteins in crude synaptosomal and enriched synaptic vesicle extracts in the L66 model of FTD.

When the phosphorylation status of transgenic human tau was examined further, we found that none of the phosphorylation-dependent monoclonal antibodies co-labeled with synapses. The labeling seen with these antibodies was restricted mainly to the cell bodies and axons of principal cells in cortex and hilus and CA1 of hippocampus. This was confirmed biochemically. Phosphorylated transgenic tau was found predominantly in low-speed supernatant fractions and remained in the supernatant following high-speed centrifugation. This was confirmed by MS sequence analysis, which identified peptides phosphorylated at Thr-181, Ser-199, Ser-202, and Thr-231 in these fractions. Of particular note, none of the peptides isolated from transgenic tau and co-purifying with synapsin-1 was found to be phosphorylated. The only exception to this was in the fraction containing crude synaptosomal constituents that sedimented with particles of size greater than 95 nm. We therefore conclude that the transgenic mutant human tau that accumulates in synapses is predominantly unphosphorylated. This protein sediments as a particle having a molecular mass of at least 200 kDa corresponding to trimeric or larger, low-order oligomers. By contrast, the aggregated tau protein found in cell bodies is largely filamentous and is found in the higher order tangle-like structures that can be labeled with Bielschowsky silver and primulin. Having this information, further immunoelectron microscopy studies may reveal more detail on the process of oligomer and filament assembly within the neuron and, particularly at the synapse.

Consistent with our findings, an association between mutant tau and synaptic vesicles has been reported previously in both *Drosophila* and rat neurons (23, 29). These vesicle-tau interactions hamper transmitter release through polymerization of N-terminal tau domains linking presynaptic F-actin with vesicle membranes, thereby making the presynaptic compartment more rigid (29). That the respective tau species destined for the presynaptic compartment can be oligomeric and nonphosphorylated was established by micropipette administration of tau through the patch pipette while recording from affected cells in the whole cell configuration (23). Voltage recordings revealed strongly reduced action potential amplitudes and slowed action potential rise and decay kinetics. Pre-synaptically, tau increased the run-down or unitary responses suggesting either reduced availability of vesicular release (29) or lowered re-uptake of transmitter in synaptic vesicles due to inhibition of P-type Ca^2+^-ATPases by tau (45, 46). At physiological pH and under challenge, these Ca^2+^ ATPases promote Ca^2+^/H^+^ exchange activity at the vesicular membrane and Ca^2+^ uptake into the vesicle (47). Neighboring cells were not affected by this treatment. Furthermore, transfection of full-length tau into neurons was associated with decreased expression of synaptic markers and reduced transport of these proteins into the presynapse (48), as well as with Ca^2+^ dysregulation (49). An association between vesicles and actin promoted through the N-terminal domain of tau (29) could contribute both to the insolubility of tau and increased rigidity in the synaptosomal compartment. Accumulation of tau in the cytoplasm is thought to interfere with a range of normal physiological processes (for review see Ref. 50). The accumulation of tau aggregates in the cytosol in L66 mice was a feature also observed in JNPL3 mice (P301L mutation). As in our L66 model, the overexpressed mutant tau led to the formation of cytoplasmic tau aggregates, neuronal loss, and motor deficits (21, 51, 52), and is consistent with what is observed in tauopathies (for review see Ref. 53).

That soluble tau species can modulate the post-synaptic compartment was suggested through the administration of intracerebroventricular recombinant or AD tau aggregates (38, 54). These investigators reported deficits in hippocampal long-term potentiation, but no deficits in paired pulse inhibition ratios. This is in line with our labeling of tau protein in fraction LP1, which contained both presynaptic (synapsin) and post-synaptic elements (PSD95). As is confirmed here for L66, these tau species are in both phosphorylated and nonphosphorylated states and require the C-terminal domain of the tau molecule (55). Some of the phosphorylation sites identified in L66 mice were also seen in WT control mice and have also been described in postmortem brain tissue of cognitively normal people, suggesting the existence of selective phosphorylation states under physiological conditions (12, 56) and are likely not related to pathological processing of tau.

The cellular distribution of different tau pools might be attributed to specific localization-dependent post-transcriptional regulation events. For example, several kinases have been described that phosphorylate tau (13, 14, 57). In addition, it has been reported that both phosphatases and kinases are also compartmentalized, and that synaptic phosphorylation activates a number of proteins (for review see Refs. 58 and 59). Therefore, it seems likely that tau undergoes compartment-specific modification and aggregation processes. A brain region-specific increase in phospho-tau-positive synaptosomes in AD patients has been reported using crude cortical synaptosomal preparations (30), similar to our MS findings in the crude P2 and LP1 factions from L66 brain tissues. Others have also reported that synaptic tau extracted from AD brain tissues is oligomeric and hyperphosphorylated (30, 31) but this has not been confirmed. Sokolow and colleagues (33) labeled the majority of synaptic terminals with HT7 (not specific for phosphorylated tau) in both AD and control subjects and found an increase in a truncated 20-kDa tau fragment lacking a C terminus and appearing as dimers in immunoblots from AD samples.

Mutant human tau was equally enriched in both supernatant and pellet after addition of urea, whereas murine tau was almost completely depleted in the urea pellet (summarized in Table 4). The urea-resistant human tau species are less likely to be membrane bound, as urea disrupts these membrane associations and hydrogen bonding within tau protein aggregates (60). Mouse tau is not insoluble in urea and remains in the supernatant. This is consistent with the observation in JNPL3 mice expressing the P301L mutation (61). The sarkosyl-insoluble preparation of tau in this model was found to be largely human and only became filamentous in older animals. It appears that the progression of tau aggregation pathology is more advanced in our L66 mice to such an extent that the filamentous state was already established at 7 months of age (21). In addition, native mouse tau was largely absent from the high-speed Triton X-100 pellet, supporting its inability to assemble into aggregates. However, it is not possible to determine from the data currently available whether there is some degree of recruitment of mouse tau into the Triton- and urea-insoluble aggregates. The mutant tau protein in the low-speed Triton X-100 supernatant was found to remain heat stable even after lengthy exposure to heat. It was also found to be stable when purified using TCA (data not shown) (62). Heat stability is an inherent property of tau as an aggregation-prone and intrinsically unstructured protein without a hydrophobic core (63, 64, 65) and is widely used as a method for separation of tau from other abundant proteins (2, 63, 64). In contrast to tau, other synaptic proteins were not heat stable. Increasing concentrations of insoluble tau have been reported to lead to formation of filamentous tau aggregates in elderly transgenic P301L tau mice (66). Triton-insoluble filamentous tau species enriched from P301S mice have a greater seeding potential *in vitro* (67) and have higher toxicity (68). However, we have reported recently that the toxicity of filamentous tau can be dissociated from the ability of truncated oligomers to induce abnormal phosphorylation of endogenous tau (69). At present, the molecular mechanism of aggregation of full-length mutant human tau remains unknown.

**Table 4:** Summary for urea/Triton X-100 solubility and heat stability of tau (see Figs. 7–9)

Experiment	Antibody	Tau Isoform	Genotype	P	S	FS^a^	FP	HP	HS
Urea	7/51	Human tau (62 kDa)	L66	2+	2+				
			WT	−	−				
		Mouse tau (55 kDa)	L66	+	2+				
			WT	+	2+				
Tris	7/51	Human tau (62 kDa)	L66	2+	2+	+	2+		
			WT	−	−	−	−		
		Mouse tau (55 kDa)	L66	2+	2+	+	2+		
			WT	2+	2+	+	2+		
Tris-Triton X-100	7/51	Human tau (62 kDa)	L66	2+	2+	2+	+		
			WT	−	−	−	−		
		Mouse tau (55 kDa)	L66	2+	+	+	+		
			WT	2+	+	+	+		
Heat stability	7/51	Human tau (62 kDa)	L66		+			+	3+
			WT		−			−	−
	HT7	Human tau (62 kDa)	L66		+			+	3+
			WT		−			−	−

a Empty cells, not applicable.

Although our data provide strong evidence that the mutant human tau that aggregates in the synaptic compartment is nonphosphorylated, this is at odds with some other reports. For example, Kimura and co-workers (66) suggest that phosphorylation is crucial for the development of synaptic tau toxicity and synapse loss in P301L mice. By contrast, Zhou *et al*. (29) reported data more in line with those reported here, confirming that synaptic function is compromised using both pathogenic human tau mutants and phosphomimetic tau species devoid of 14 putative phosphor-serine/threonine residues (changed to glutamate residues). These data are consistent with evidence showing that tau aggregation and toxicity can both occur in the absence of any phosphorylation (16, 22, 69). Phosphorylation of tau inhibits tau–tau binding (16, 17) and might play a protective role against its toxicity (17, 70, 71, 72, 73). Consistent with this, aggregation of nonphosphorylated tau correlates with the onset and extent of cognitive impairment in mice (21) and humans (74, 75), whereas phospho-tau pathology appears to be a late stage event in the disease progression (74). It is not known at present what triggers pathological compartmentalization of tau and how specifically this affects synaptic membranes or vesicles.

Although we have gone some way toward defining the abnormal compartmentalization of transgenic human tau in a mouse model of FTD, there are important limitations in the work to date. The tau protein accumulating in the core of the tau filaments found in FTD brain tissues is restricted to the repeat region similar to that found in the core of the PHF in AD (3, 76, 77), albeit with a different fold (78). This core is proteolytically stable and propagates the aggregation cascade via a high affinity tau–tau binding interaction (16, 79). The present study has focused on the soluble transgenic mutant tau species that can be isolated from mouse brain. The tau protein that remains insoluble after urea and/or Triton X-100 treatment needs to be characterized further with a view to determining the identity of any proteolytically stable and insoluble tau species generated in this model. In L66 mice, the human tau is expressed in a form that readily forms insoluble aggregates that do not incorporate endogenous murine tau. Evidence for incorporation of endogenous tau into tau aggregates in mice expressing different isoforms of human tau is variable. Although no co-aggregation was seen in similar tau P301L mice (28, 61, 80), others have reported such co-aggregation, *e.g.* in TauΔK280 mice (81, 82, 83, 84). Further models, such as those expressing 3R isoforms, may differ and remain to be tested.

In summary, we here present an attempt to determine the physicochemical changes of tau protein in different cellular compartments in the L66 mouse model for FTD. We show that transgenic mutant human tau form distinct insoluble aggregates that do not incorporate endogenous mouse tau. The 62-kDa human tau forms larger aggregates that are resistant to urea and Triton X-100. Other species, oligomers of smaller size that were sedimented at higher speed, were partially soluble after Triton X-100 treatment. The tau species that concentrate in synapses are in a nonphosphorylated form. Another pool of tau, which is in part Triton X-100–soluble, is heat stable, phosphorylated, and presumably cytosolic. Native mouse tau, on the other hand, does not accumulate in synaptic fractions and is neither urea/Triton X-100–resistant nor heat stable. The analyses presented here for an FTD model, when compared with physiologically appropriate AD models, will shed light on how different tau conformations develop. This may help understand the distinct pathologies observed for different tauopathies and provide new therapeutic targets that may be either shared between tauopathies or unique to individual disorders.

## Experimental procedures

### Animals

Tau transgenic L66 and NMRI WT control mice were used in this study and have been described in detail previously (21). L66 mice overexpress the longest human tau isoform (2N4R) with 441 amino acid residues, under control of the mouse *Thy1*-promoter. Two aggregation-promoting mutations, P301S and G335D in the repeat domain, were inserted into the tau cDNA and this mutated isoform is hereafter termed Tau-X. Female homozygous transgenic L66 (*n* = 15) and WT litters (*n* = 12) were bred commercially (Charles River Laboratories, UK) in isolators and delivered by truck to Aberdeen at least 10 days before experimental work commenced. They were housed by genotype in small colonies up to 5 animals in open housing (Macrolon III) with corncob bedding and paper strips and cardboard tubes as enrichment (cleaning rota once per week). Holding rooms were on constant temperature (20-22°C), humidity (60–65%), and air exchange rate (17-20 changes/h) with 12 h light/dark cycle (lights on at 6 am, simulated dawn). Animals had free access to food and water and were aged 6-7 months when sacrificed by cervical dislocation. Brains were rapidly extracted and snap frozen in liquid nitrogen for protein studies or fixed for 24 h in formalin and embedded in paraffin for histopathology. There was no blinding or randomization at this stage of the experiment. All animal experiments were performed in accordance with the European Communities Council Directive (63/2010/EU) and a project license with local ethical approval under the UK Animals (Scientific Procedures) Act (1986). Brains were sent to Berlin via courier on dry ice.

### Immunohistochemistry and immunofluorescence

Five-µm coronal sections were collected at desired brain areas (corresponding to sections Bregma −3.8 mm for hippocampus and auditory/visual cortices and Bregma +0.74 mm for striatum and motor/entorhinal cortices of the mouse brain atlas (85)) from randomly chosen L66 (*n* = 5) and WT (*n* = 5) mice, de-waxed, boiled in 10 mm citrate buffer, and further processed for immunohistochemistry or immunofluorescence as described below.

For immunohistochemistry, sections were boiled in 10 mm citric buffer, incubated in hydrogen peroxidase solution (0.3% (v/v)) for 10 min, and then for 20 min in blocking buffer (0.1% (w/v) BSA in PBS). Sections were incubated in primary and secondary antibodies diluted in blocking buffer, each for 1 h at room temperature with 3× 10 min PBS-washing steps after each antibody. Sections were developed with diaminobenzidine solution (Dako, Denmark) and embedded in Neo-Mount® (Merck Millipore, Germany). Anti-tau antibodies directed against both phosphorylated and nonphosphorylated tau protein, and with epitopes in N- and C-terminal domains were used: mAb 7/51, Tau46, HT7, mAb 27/499, pSer-202, pSer-396, and pSer-416. The epitopes recognized by these antibodies are illustrated in Fig. 1 and their details listed in Table 1.

For immunofluorescence, sections were blocked for 1 h in blocking buffer (5% (v/v) normal goat serum in PBS containing 0.3% (v/v) Triton X-100) and incubated overnight at 4 °C in primary antibody mixture (HT7 and synapsin-2; pSer-202 and synapsin-2, pSer-396 and synapsin-1 as well as pSer-416 and synapsin-2, see Table 1 for dilutions) diluted in blocking buffer. The next day, sections were washed 3× 10 min with PBS, incubated for 1.5 h in fluorochrome-conjugated secondary antibodies (Alexa Fluor® 488-conjugated donkey anti-mouse IgG and Alexa Fluor® 568-conjugated goat anti-rabbit IgG, Life Technologies, USA; both diluted 1:500 in blocking buffer), washed again 3× 10 min with PBS, covered with DAPI Gold Antifade Reagent (Cell Signaling Technology, MA, USA), and examined using a microscope equipped for fluorescence (Carl Zeiss, Jena, Germany).

### Subcellular fractionation of brain tissues

Subcellular fractionation was conducted as described before (34) and the workflow is shown in Fig. 4. Randomly selected brains from L66 (*n* = 5) and WT (*n* = 4) mice were used. Briefly, 10 volumes of Tris buffer (25 mm, pH 7.4) containing 9% sucrose, 2 mm EDTA, 5 mm DTT and protease and phosphatase inhibitors were added to crushed frozen brain tissues (pulverized using a pestle and mortar with liquid nitrogen) and repeatedly pipetted up and down. The homogenate was centrifuged for 5 min at 4 °C and 1,000 × *g* to obtain a supernatant (S1) and pellet (P1, containing nuclei and large debris). S1 was centrifuged for 15 min at 4 °C and 12,500 × *g* to obtain a further supernatant (S2, see below) and a crude synaptosomal fraction pellet (P2). P2 was subsequently lysed in ice-cold water and the lysate adjusted to 25 mm Tris and incubated for 30 min at 4 °C. Thereafter, P2 was centrifuged at 25,000 × *g* for 20 min at 4 °C to pellet a pre- and postsynaptic membrane fraction (LP1). The supernatant fraction (LS1) was further centrifuged for 2 h at 4 °C and 176,000 × *g* to obtain a synaptic vesicle pellet fraction (LP2) and a supernatant fraction (LS2). The S2 fraction from above was centrifuged at 176,000 × *g* for 1 h at 4 °C to obtain a cytosolic fraction (S3) and a light membrane/microsome-enriched pellet fraction (P3). The pellets (P1, P3, LP1, and LP2) were each suspended in 1 volume of reducing Laemmli buffer (125 mm Tris-HCl, pH 7.0, 0.8% (w/v) SDS, 20% (v/v) glycerol, and 10% (v/v) 2-mercaptoethanol). Protein concentration was determined using the Bradford reagent (Carl Roth, Karlsruhe, Germany), according to the manufacturer's recommendations.

### Protein extraction from brain tissue using urea

Crushed frozen brain tissue from L66 (*n* = 5) and WT (*n* = 3) mice was incubated for 45 min at room temperature in 6 volumes of urea extraction buffer (7 m urea, 2 m thiourea, 2% ampholyte 2-4, 70 mm DTT, 25 mm Tris/HCl, pH 8.0, 50 mm KCl, 3 mm EDTA, 2.9 mm benzamidine, and 2.1 μm leupeptin) and separated by centrifugation at 16,000 × *g* for 45 min at room temperature. The supernatant (S) was transferred to new tubes. The pellet was mixed with 1 volume of reducing Laemmli buffer, boiled for 5 min at 95 °C, and the soluble pellet (P) was retained for later analysis. Both S and P fractions were used for tau characterization. The protein concentration of all fractions was determined as stated above.

### Protein extraction from brain tissue with and without detergent

Five volumes of Tris extraction buffer (30 mm Tris, pH 7.4, containing cOmplete™ protease inhibitor and PhosStop™ phosphatase inhibitor mixture tablets from Roche), with or without the addition of Triton X-100 (0.1% (v/v)), were added to crushed frozen tissue (5 L66 and 3 WT brains pulverized using pestle and mortar with liquid nitrogen), repeatedly pipetted up and down for homogenization and incubated for 30 min at 4 °C. The extraction procedure is shown in Fig. 9*A*. The homogenate was centrifuged for 30 min at 16,000 × *g* and 4 °C. The supernatant (S) was transferred to a new tube and the pellet (P) suspended in 1 volume of reducing Laemmli buffer. Some of the supernatant (S) was retained for analysis, whereas the remaining supernatant was centrifuged for 1 h at 100,000 × *g* and 4 °C. The resulting high-speed supernatant (FS) was transferred into a new tube and the high-speed pellet (FP) mixed with 1 volume of reducing Laemmli buffer.

### Heat denaturation

For heat denaturation, low-speed supernatant (S) from the above-mentioned Tris extraction, was boiled for either 5 or 30 min at 95 °C and the reaction stopped by transfer to ice. The boiled mixture was subjected to centrifugation at 16,000 × *g* for 5 min to separate supernatant fraction (HS, which is heat-stable) from heat-pelleted fraction (HP) and the latter was suspended with 1 volume of reducing Laemmli buffer. The protein concentration of all fractions was determined using the Bradford method.

### One-dimensional electrophoresis

Samples derived from urea extraction, detergent extraction, heat denaturation, and subcellular fractionation were subjected to Tricine or glycine one-dimensional electrophoresis. Loading details are given in the legends of Figure 5, Figure 6, Figure 7, Figure 8, Figure 9, Figure 10. The Tricine gels (10%) were run using a discontinuous buffer. The cathode buffer consisted of 100 mm Tris, 100 mm Tricine, and 1% (w/v) SDS and the anode buffer contained 100 mm Tris and 0.07% (v/v) HCl. The glycine gels (10% gels or commercial gradient gels from 4–15 or 4–20%, Bio-Rad Laboratories, USA) were run in Tris glycine buffer containing 192 mm glycine, 25 mm Tris, and 0.9% (w/v) SDS.

### MS compatible silver staining

Gels were fixed overnight in 50% (v/v) ethanol, 10% (v/v) acetic acid and thereafter silver stained according to standard procedures (86). Briefly, gels were rinsed for 10 min in 20% (v/v) ethanol, incubated for 1 min in 0.02% (w/v) sodium thiosulfate solution, and rinsed twice in water. Then gels were incubated for 30 min in 0.1% (w/v) silver nitrate solution, rinsed for 1 min in water followed by 3-6 min incubation in developing solution (2.5% (w/v) sodium carbonate, 0.02% (v/v) formaldehyde, 0.025% (w/v) thimerosal). The reaction was stopped by 2× 10 min incubation in 1.85% (w/v) EDTA disodium salt dihydrate, Titration complex III. These gels were used to excise spots for MS analysis.

### Protein immunoblotting

Proteins from glycine gels were transferred to polyvinylidene difluoride membranes at U_const_ = 5 V in Towbin transfer buffer (25 mm Tris, 200 mm glycine, 0.1% (w/v) SDS, and 20% (v/v) ethanol) for 30 min, whereas proteins from tricine electrophoresis were transferred to polyvinylidene difluoride at 4 °C and *I*_const_ = 0.4 mA/cm^2^ in tricine transfer buffer (300 mm tricine, 6% (v/v) acetic acid, pH 8.6) overnight. Afterward, membranes were blocked for 1 h in blocking solution (4% (w/v) BSA in TBS with 0.2% (v/v) Tween-20), incubated overnight at 4 °C in primary antibody, diluted in blocking solution, washed 3 times in TBS-T (TBS with 0.2% (v/v) Tween-20), and incubated for 1 h in secondary antibody (Dako, Denmark), diluted 1:5,000 in blocking solution. After washing 3 more times in TBS-T, membranes were overlaid with ECL solution (100 mm Tris, pH 8.5, 1.25 mm luminol, 200 μm *p*-coumaric acid, 0.01% (v/v) H_2_O_2_) and chemiluminescence signals were detected on hyper-films (GE Healthcare, USA) or by the ChemiDoc Imaging System (Bio-Rad Laboratories, USA). All incubations were conducted at room temperature, unless otherwise stated. The antibodies tested were mAb 7/51, HT7, Tau46, β-actin, SNAP25, synapsin-1, syntaxin-1, and PSD95 and details are given in Table 1. A tau ladder (T7951, Sigma, USA) comprising all six human tau isoforms was included as control for anti-tau immunoreactivity. Signals were densitometrically quantified using the AlphaEase software version 3.1.2 (Alpha Innotech Corporation, USA) or Image Laboratory software version 5.0 (Bio-Rad Laboratories, USA) and values plotted as group mean ± S.E., without intention to perform statistical analyses.

### MS analysis and protein identification criteria

Spots of interest were excised from silver-stained gels and excised gel bands were prepared for enzymatic cleavage by 3 times swelling/shrinking in 100 mm aqueous tetraethylammonium bicarbonate solution or 50 mm tetraethylammonium bicarbonate in 60% acetonitrile, respectively. For reduction and alkylation of cysteine residues, the bands were treated with tris(2-carboxyethyl)phosphine (5 mm final) and iodoacetamide (5 mm final) during consecutive swelling steps. Each step was carried out for 30 min at room temperature. After the last shrinking step, the gel slices were dried for 5 min in open Eppendorf tubes. Proteolysis was conducted by addition of trypsin or thermolysin (200 ng/spot) and overnight incubation at 37 °C. The resulting peptides were acidified with formic acid (0.5% (v/v) final concentration) prior to mass spectrometric analysis. Peptides were analyzed by nanoLC-ESI-MS/MS. The LC–MS system consisted of an Agilent 1100 nanoHPLC system (Agilent, Germany), a PicoTip electrospray emitter (New Objective, USA) and an Orbitrap XL mass spectrometer (ThermoFisher, Bremen, Germany). After trapping and desalting the peptides on a Zorbax 300SB-C18 enrichment column (0.3 mm × 5 mm, Agilent, Germany) for 5 min using 0.5% (v/v) formic acid solution, peptides were separated on a Zorbax 300-SB-C18 column (75 μm × 150 mm, Agilent, Germany) within 35 min using an acetonitrile, 0.1% formic acid gradient from 15 to 40% acetonitrile. The Orbitrap instrument was operated in a data-dependent mode by subjecting the 10 most abundant ions of each survey spectrum (nominal resolution 35,000) to CID fragmentation, with a normalized collision energy set at 35%. MGF files were generated by DTA SuperCharge (version 2.0, RRID:SCR_019206) and the MS/MS Mascot search engine version 2.2 (Matrix Science, UK) was used for protein identification. A custom protein database was used comprising a complete UniProtKB *Mus musculus* protein database (with 24728 entries, downloaded from RRID:SCR_002380 on December 2015), into which we introduced the 6 human WT tau isoforms Tau-A to Tau-F (downloaded from RRID:SCR_002380 on December 2015) and the mutant tau isoform from L66 mice, named Tau-X (equivalent to Tau-F (2R4N) with the addition of P301S and G335D mutations). The following search parameters for Mascot were used and no additional threshold was applied (all significant hits with *p* < 0.05 were considered): (i) instrument: ion trap: (ii) enzyme: trypsin and thermolysin, 2 missed cleavages allowed; (iii) variable modifications: acetyl (N-term), oxidation (M), and deamidation (NQ); (iv) fixed modifications: carbamidomethyl (C); (v) mass values: monoisotopic; (vi) fragment search tolerance: 2 ppm for peptide mass and 0.5 Da; (vii) peptide charge: 1+, 2+ and 3+. For identification of phosphorylated Tau-X species, PEAKS software (version 8.0, Bioinformatics Solutions Inc.) was used with the following settings: variable modifications: carbamidomethylation, propionamide, oxidation (M), deamidation (NQ), and phosphorylation (STY), precursor ion tolerance: 3 ppm, fragment ion tolerance: 0.6 Da, peptide hit threshold (−10 logP) ≥ 20.0, FDR < 0.1% (FDR was calculated by the decoy fusion method as described in Zhang (87)), and maximum number of PTMs: 5.

## Data availability

All data are provided within the manuscript and under the Supporting information. Raw MS files are the accessible on the MassIVE database, accession number MSV000085945.

WisTa Laboratories LtdValeria Melis, Dilyara Lauer, Mandy Magbagbeolu, Charles R. Harrington, Gernot Riedel, Claude M. Wischik, Franz Theuring, and Karima Schwab
European Union Horizon 2020 Research (15HLT02 ReMiND) to Nora Lemke, and Franz Theuring
