# Supplementary material for: Differential compartmental processing and phosphorylation of pathogenic human tau and native mouse tau in the line 66 model of frontotemporal dementia
Source: J Biol Chem. 2021 Jan 13;295(52):18508–23. doi: 10.1074/jbc.RA120.014890 (PMC7939472; doi:10.1074/jbc.RA120.014890)

Supporting Information

Differential compartmental processing and phosphorylation of pathogenic human tau and native mouse tau in the Line 66 model of frontotemporal dementia

Nora Lemke[1,2], Valeria Melis[3], Dilyara Lauer[1], Mandy Magbagbeolu[1], Boris Neumann[1,4], Charles R. Harrington[3,5], Gernot Riedel[3], Claude M. Wischik[3,5], Franz Theuring[1], Karima Schwab[1*]

[1] Charité - Universitätsmedizin Berlin, Hessische Str. 3-4, 10115 Berlin, Germany

[2] Bundesanstalt für Materialforschung und-prüfung, Richard-Willstätter-Str. 11, 12489 Berlin, Germany

[3] School of Medicine, Medical Sciences and Nutrition, University of Aberdeen, Foresterhill, Aberdeen, AB25 2ZD, UK

[4] Proteome Factory AG, Magnusstr. 11, 12489 Berlin, Germany

[5] TauRx Therapeutics Ltd., 395 King Street, Aberdeen, AB24 5RP, UK

*Corresponding author: Karima Schwab

Email: karima.schwab@charite.de

Supplementary Tables

Table S1: : Identification of immuno-reactive species S1, P2, S2, P3, S3, LP1, LS1, LP2 and LS2 by Orbitrap LC-MS and Mascot search engine. The bands at a size of around 62 kDa were excised from preparative silver gels (see Fig. 5 for fractions). MAPT: microtubule-associated protein tau. Human tau isoforms (Tau-A to Tau-F) were identified by database search, nomenclature according to UniProt database. Although high sequence homology does not permit excluding mouse tau from the table, mouse species are not shown here as mouse tau is present at 55 kDa and not at 62 kDa. Furthermore, due to the high sequence homology, transgenic tau isoform Tau-X was only identified unequivocally when the sequence coverage was high enough. Peptide and protein score (-10Log(p)) is the measure of significance. Peptide mass error: deviation of the measured peptide mass from the theoretical peptide mass. ppm: parts per million. Regular font: peptides derived from tryptic digest, italics: peptides derived from thermolysin digest, double dagger (‡): thermolysin peptide which was also identified in tryptic digest.

Fraction	Position of tau in hit list	Protein isoform (Sequence coverage)	Number of AA	Protein score	Number of peptides matched	Matched peptide	m/z	Pre-cursor charge	Peptide score	Peptide mass error [ppm]	Number of missed peptide cleavages
S1	15	Tau-X (49%)	441	809	18	LDLSNVQSK	502.2762	+2	52	3.27	0
						TPSLPTPPTR	533.7976	+2	31	-1.14	0
						KLDLSNVQSK	566.3218	+2	71	-0.36	1
						SEKLDFKDR	379.8684	+3	28	0.81	2
						LQTAPVPMPDLK	655.3640	+2	56	1.73	0
						SGYSSPGSPGTPGSR	697.3212	+2	80	0.65	0
						TPSLPTPPTREPK	710.8942	+2	42	1.12	1
						IGSLDNITHVPGGGNK	526.9476	+3	40	3.13	0
						VAVVRTPPKSPSSAK	535.2905	+3	33	0.12	2
						TPPAPKTPPSSGEPPK	556.6054	+3	42	-1.5	1
						IGSLDNITHVPGGGNKK	569.6445	+3	36	0.29	1
						STPTAEDVTAPLVDEGAPGK	977.9868	+2	67	3.06	0
						HVSGGGSVQIVYKPVDLSK	657.3614	+3	57	-0.44	0
						CGSLGNIHHKPGGDQVEVK	678.0048	+3	33	-1.53	0
						QEFEVMEDHAGTYGLGDR	685.3001	+3	48	-3.09	0
						TPPAPKTPPSSGEPPKSGDR	695.0000	+3	32	-0.08	2
						HLSNVSSTGSIDMVDSPQLATLADEVSASLAK	1087.2088	+3	63	3.71	0
						‡ LQTAPVPMPDLK	*655.3627*	*+2*	*64*	*-0.19*	*3*
						LDNITHVPGGGNKK	*483.9317*	*+3*	*37*	*-0.25*	*4*
P2	31	Tau-X (12%)	441	237	4	KLDLSNVQSK	566.3206	+2	52	-2.57	1
		Tau-D (17%)	383			LQTAPVPMPDLK	655.3635	+2	56	1.06	0
		Tau-E (16%)	412			SGYSSPGSPGTPGSR	697.3198	+2	88	-1.33	0
		Tau-F (14%)	441			IGSLDNITHVPGGGNK	526.9461	+3	43	0.28	0
S2	20	Tau-X (45%)	441	777	17	LDLSNVQSK	502.2754	+2	55	1.7	0
						TPSLPTPPTR	533.7975	+2	46	-1.41	0
						KLDLSNVQSK	566.3227	+2	74	1.21	1
						SEKLDFKDR	379.8688	+3	40	1.89	2
						LQTAPVPMPDLK	663.3599	+2	54	-0.65	0
						SGYSSPGSPGTPGSR	697.3209	+2	94	0.19	0
						TPSLPTPPTREPK	474.2649	+3	30	0.49	1
						IGSLDNITHVPGGGNK	526.9470	+3	37	1.87	0
						TPPAPKTPPSSGEPPK	556.6059	+3	33	-0.48	1
						IGSLDNITHVPGGGNKK	569.6441	+3	37	-0.34	1
						STPTAEDVTAPLVDEGAPGK	977.9851	+2	82	1.3	0
						HVSGGGSVQIVYKPVDLSK	657.3619	+3	38	0.35	0
						CGSLGNIHHKPGGDQVEVK	678.0058	+3	31	-0.08	0
						QEFEVMEDHAGTYGLGDR	685.3035	+3	54	1.87	0
						HLSNVSSTGSIDMVDSPQLATLADEVSASLAK	1087.2060	+3	74	1.13	0
						‡ LQTAPVPMPDLK	*655.3623*	*+2*	*63*	*-0.78*	*3*
						FEVMEDHAGTYG	*686.2813*	*+2*	*44*	*2.28*	*2*
						IGDTPSLEDEAAGH	*706.3202*	*+2*	*74*	*-0.36*	*3*
P3	21	Tau-X (41%)	441	782	14	LDLSNVQSK	502.2768	+2	41	4.52	0
						KLDLSNVQSK	566.3233	+2	72	2.15	1
						SEKLDFKDR	379.8679	+3	39	-0.5	2
						LQTAPVPMPDLK	663.3611	+2	57	1.12	0
						SGYSSPGSPGTPGSR	697.3212	+2	84	0.55	0
						TPSLPTPPTREPK	474.2662	+3	30	3.34	1
						IGSLDNITHVPGGGNK	526.9470	+3	54	1.89	0
						IGSLDNITHVPGGGNKK	569.6427	+3	42	-2.9	1
						STPTAEDVTAPLVDEGAPGK	977.9867	+2	84	2.93	0
						HVSGGGSVQIVYKPVDLSK	657.3623	+3	62	0.97	0
						CGSLGNIHHKPGGDQVEVK	678.0070	+3	41	1.74	0
						QEFEVMEDHAGTYGLGDR	685.3018	+3	45	-0.51	0
						QEFEVMEDHAGTYGLGDRK	546.2526	+4	37	0.8	1
						HLSNVSSTGSIDMVDSPQLATLADEVSASLAK	1087.2063	+3	96	1.36	0
						‡ LQTAPVPMPDLK	*655.3633*	*+2*	*53*	*0.75*	*3*
S3	20	Tau-X (45%)	441	782	16	LDLSNVQSK	502.2757	+2	61	2.3	0
						TPSLPTPPTR	533.7997	+2	31	2.88	0
						KLDLSNVQSK	566.3230	+2	67	1.73	1

						SEKLDFKDR	379.8685	+3	30	0.88	2
						LQTAPVPMPDLK	663.3599	+2	63	-0.67	0
						SGYSSPGSPGTPGSR	697.3206	+2	79	-0.19	0
						IGSLDNITHVPGGGNK	526.9471	+3	53	2.12	0
						TPPAPKTPPSSGEPPK	556.6057	+3	37	-0.95	1
						IGSLDNITHVPGGGNKK	569.6453	+3	45	1.64	1
						STPTAEDVTAPLVDEGAPGK	977.9855	+2	77	1.74	0
						HVSGGGSVQIVYKPVDLSK	657.3623	+3	52	0.85	0
						CGSLGNIHHKPGGDQVEVK	678.0057	+3	35	-0.19	0
						QEFEVMEDHAGTYGLGDR	685.3022	+3	67	0.05	0
						TPPAPKTPPSSGEPPKSGDR	694.9981	+3	31	-2.72	2
						HLSNVSSTGSIDMVDSPQLATLADEVSASLAK	1087.2050	+3	55	0.22	0
						‡ LQTAPVPMPDLK	*655.3624*	*+2*	*54*	*-0.69*	*3*
						FEVMEDHAGTYG	*686.2808*	*+2*	*48*	*1.54*	*2*
LP1	21	Tau-X (35%)	441	625	13	LDLSNVQSK	502.2742	+2	52	-0.65	0
						TPSLPTPPTR	533.7994	+2	29	2.16	0
						KLDLSNVQSK	566.3227	+2	46	1.1	1
						LQTAPVPMPDLK	663.3607	+2	66	0.59	0
						SGYSSPGSPGTPGSR	697.3201	+2	76	-1.01	0
						IGSLDNITHVPGGGNK	526.9467	+3	54	1.4	0
						TPPAPKTPPSSGEPPK	556.6054	+3	41	-1.5	1
						IGSLDNITHVPGGGNKK	569.6443	+3	42	0.02	1
						STPTAEDVTAPLVDEGAPGK	977.9810	+2	91	-2.95	0
						HVSGGGSVQIVYKPVDLSK	657.3616	+3	39	-0.15	0
						CGSLGNIHHKPGGDQVEVK	678.0059	+3	36	0.07	0
						QEFEVMEDHAGTYGLGDR	685.3023	+3	53	0.2	0
						‡ LQTAPVPMPDLK	*663.3608*	*+2*	*49*	*0.79*	*3*
						FEVMEDHAGTYG	*686.2797*	*+2*	*45*	*-0.06*	*2*
LS1	30	Tau-X (14%)	441	330	6	LDLSNVQSK	502.2749	+2	43	0.68	0
		Tau-D (16%)	383			TPSLPTPPTR	533.7988	+2	36	1.18	0
		Tau-E (15%)	412			KLDLSNVQSK	566.3224	+2	67	0.58	1
		Tau-F (14%)	441			LQTAPVPMPDLK	655.3634	+2	66	0.78	0
						SGYSSPGSPGTPGSR	697.3205	+2	73	-0.41	0
						IGSLDNITHVPGGGNK	526.9459	+3	47	-0.2	0
LP2	43	Tau-X (9%)	441	149	3	LQTAPVPMPDLK	655.3626	+2	51	-0.39	0
		Tau-Fetal (12%)	352			SGYSSPGSPGTPGSR	697.3204	+2	63	-0.55	0
		Tau-A (13%)	316			IGSLDNITHVPGGGNK	526.9457	+3	37	-0.5	0
		Tau-B (11%)	381								
		Tau-C (10%)	410								
		Tau-D (11%)	383								
		Tau-E (10%)	412								
		Tau-F (9%)	441								
LS2	59	Tau-X (3%)	441	61	1	SGYSSPGSPGTPGSR	697.3205	+2	61	-0.37	0
		Tau-Fetal (4%)	352								
		Tau-A (4%)	316								
		Tau-B (3%)	381								
		Tau-C (3%)	410								
		Tau-D (3%)	383								
		Tau-E (3%)	412								
		Tau-F (3%)	441								

Table S2: Identification of phosphorylated Tau-X species derived from subcellular fractionation (see Fig. 5) by Orbitrap LC-MS and PEAKS software analysis. Peptide score (-10Log(p)) is the measure of significance. Peptide mass error: deviation of the measured peptide mass from the theoretical peptide mass. ppm: parts per million, AA: amino acid, S: Serine, T: Threonine, +79.97: mass of phosphorylation (monoisotopic).

Fraction	Number of peptides matched (PEAKS)	Sequence coverage	Number of phospho-peptides matched	Matched phosphorylated peptide	Position of phosphorylation (AA)	Peptide score (-10log(p))	Peptide mass	Peptide mass error (ppm)
S1	21	45%	4	SGYSSPGS(+79.97)PGTPGSR	S202	55.03	1472.5933	-0.2
				TPPAPKT(+79.97)PPSSGEPPK	T181	47.11	1666.7966	-0.5
				TPPAPKT(+79.97)PPSSGEPPKSGDR	T181	37.04	2081.9783	-0.9
				VAVVRT(+79.97)PPKSPSSAK	T231	27.30	1602.8494	-0.3
P2	5		no phosphorylated tau peptides found					
S2	28	53%	6	SGYSSPGS(+79.97)PGTPGSR	S202	56.04	1472.5933	-0.8
				SGYSS(+79.97)PGSPGTPGSR	S399	54.98	1472.5933	0.6
				TPPAPKT(+79.97)PPSSGEPPK	T181	52.52	1666.7966	1.1
				TPPAPKT(+79.97)PPSSGEPPKSGDR	T181	42.53	2081.9783	-0.8
				VAVVRT(+79.97)PPKSPSSAK	T231	32.59	1602.8494	-1.0
				KVAVVRT(+79.97)PPKSPSSAK	T231	29.68	1730.9443	-0.2
P3	14	42%	no phosphorylated tau peptides found					
S3	22	50%	4	TPPAPKT(+79.97)PPSSGEPPK	T181	45.13	1666.7966	0.3
				TPPAPKT(+79.97)PPSSGEPPKSGDR	T181	41.25	2081.9783	-1.5
				VAVVRT(+79.97)PPKSPSSAK	T231	27.96	1602.8494	-0.2
				KVAVVRT(+79.97)PPKSPSSAK	T231	26.61	1730.9443	0.0
LP1	20	32%	4	SGYSSPGS(+79.97)PGTPGSR	S202	47.03	1472.5933	-0.4
				TPPAPKT(+79.97)PPSSGEPPK	T181	28.49	1666.7966	-0.7
				KVAVVRT(+79.97)PPKSPSSAK	T231	25.22	1730.9443	0.0
				TPPAPKT(+79.97)PPSSGEPPKSGDR	T181	23.01	2081.9783	-1.4
LS1	6	11%	no phosphorylated tau peptides found					
LP2	2	6%	no phosphorylated tau peptides found					
LS2	1	3%	no phosphorylated tau peptides found					

Supplementary Figures

Figure S1: Tau immunohistochemistry in L66 and WT control mice. Tau immunoreactivity with phosphorylation-independent antibodies 7/51 (A), Tau46 (B), HT7 (C) and 27/499 (D), and with phosphorylation-specific antibodies pSer202 (E), pSer396 (F) and pSer416 (G) show prominent intraneuronal staining in cortex and hippocampus in L66 brains. Phosphorylation-independent antibodies showed strong synaptic staining mainly in striatum in L66 (A-D). Phosphorylation-specific antibodies showed strong staining in cell bodies and axons in L66 (E-G). Black arrowhead: cytosolic staining in neurons, white arrowhead: axonal/dendritic staining, circle: synaptic staining. CA1: hippocampus subfield CA1, STRI: striatum, ERC: entorhinal cortex. Scale bars, 100 µm. L66: Line 66 tau transgenic mice. WT: wild-type mice.

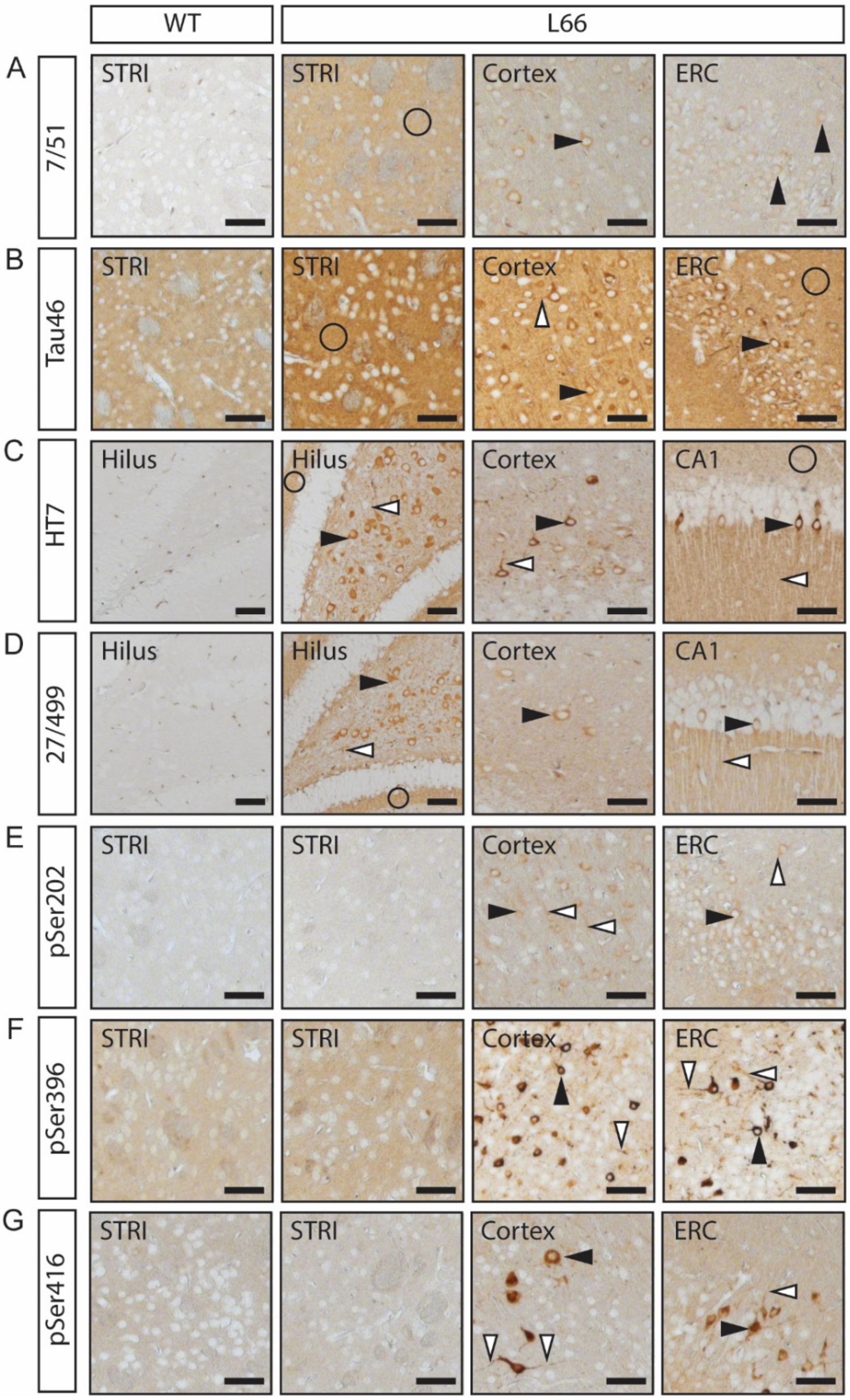

Figure S2: Co-staining for synapsin and phospho-tau in L66 mice, using immunofluorescence. Tau immunoreactivity with the phosphorylation-dependent antibodies pSer202, pSer396 or pSer416 (green) with synapsin-1 or synapsin-2 (red). In the merged panels on the right: arrows indicate tau staining in the neuronal soma; double arrowheads, synaptic tau. Scale bars, 25 µm. Phospho-tau is present in the cell soma but is virtually absent from synapses.

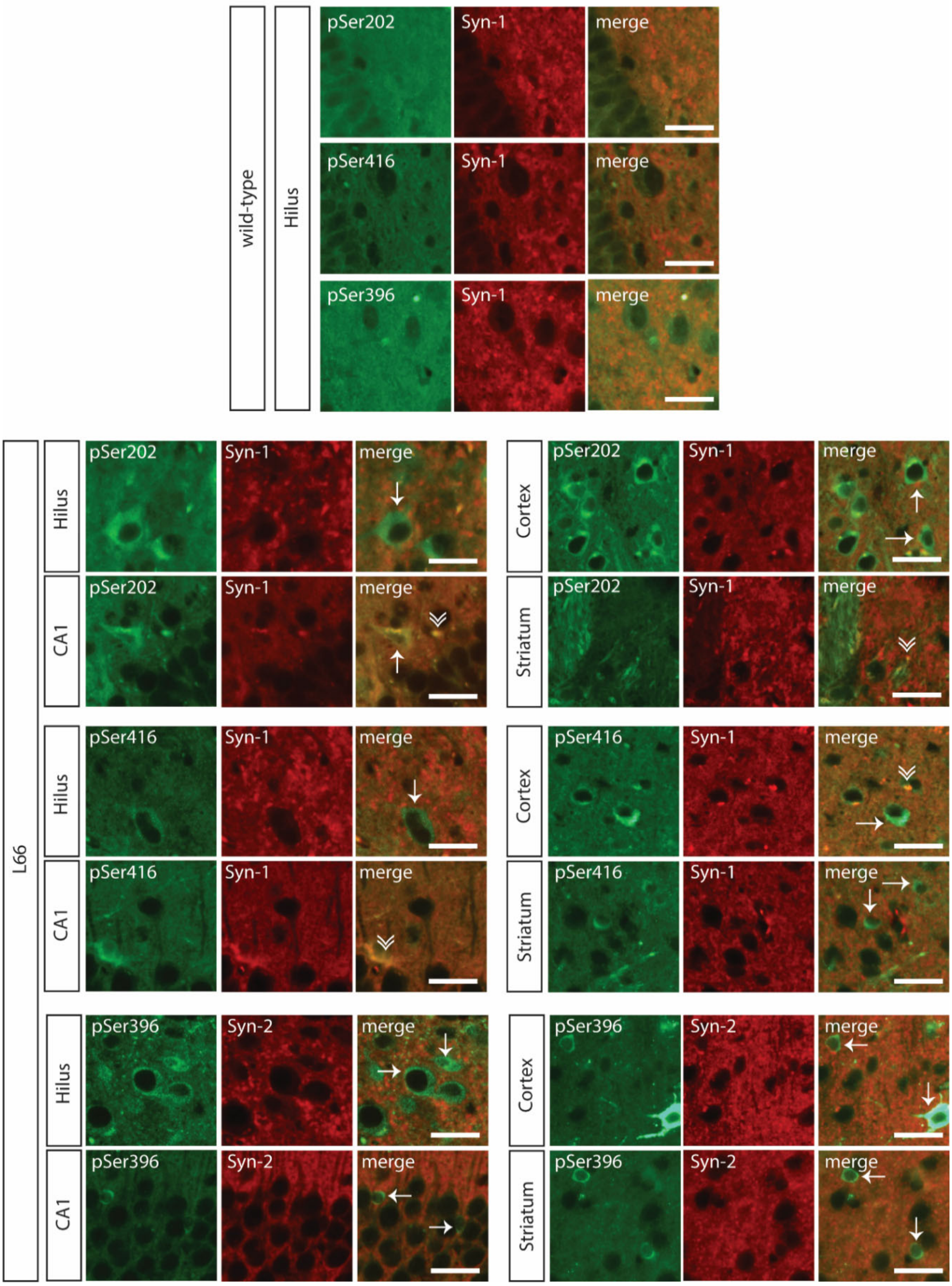

Figure S3: Annotated MS/MS spectrum of single peptide identification of Tau-X species derived from subcellular fractionation in fraction LS2 (see Table S1) by Orbitrap LC-MS and Mascot software analysis. Peptide SGYSSPGSPGTPGSR, mass 1392.5933

Annotated spectrum with alignment

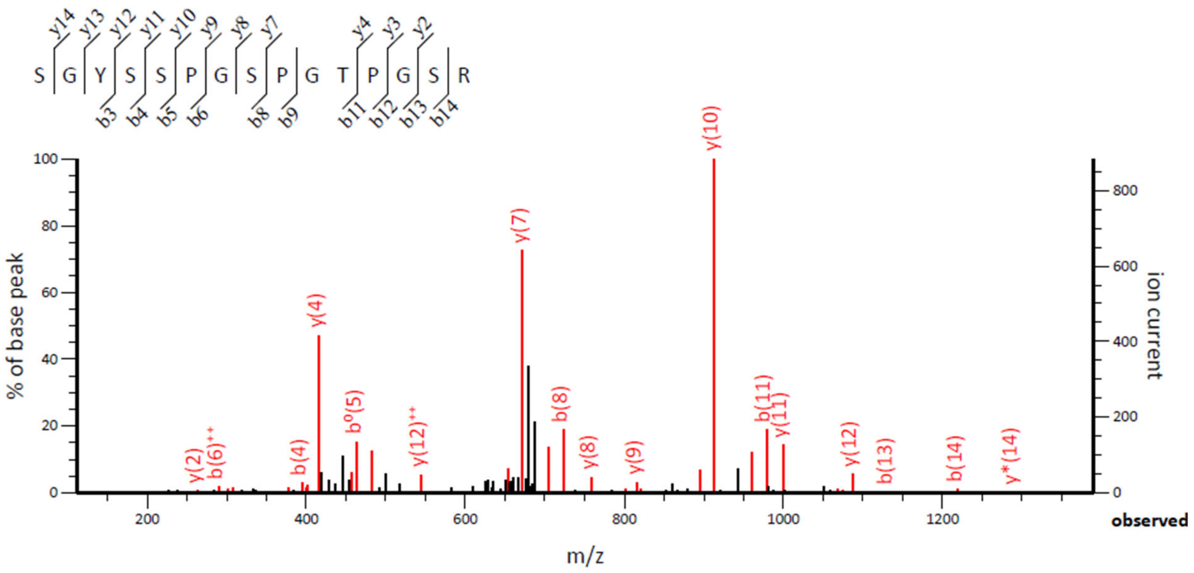

Ion table and mass error map

#	b	b++	b0	b0++	Seq.	y	y++	y*	y*++	y0	y0++	#
1	88.0393	44.5233	70.0287	35.5180	S							15
2	145.0608	73.0340	127.0502	64.0287	G	1306.6022	653.8047	1289.5757	645.2915	1288.5917	644.7995	14
3	308.1241	154.5657	290.1135	145.5604	Y	1249.5808	625.2940	1232.5542	616.7807	1231.5702	616.2887	13
4	395.1561	198.0817	377.1456	189.0764	S	1086.5174	543.7624	1069.4909	535.2491	1068.5069	534.7571	12
5	482.1882	241.5977	464.1776	232.5924	S	999.4854	500.2463	982.4588	491.7331	981.4748	491.2411	11
6	579.2409	290.1241	561.2304	281.1188	P	912.4534	456.7303	895.4268	448.2170	894.4428	447.7250	10
7	636.2624	318.6348	618.2518	309.6295	G	815.4006	408.2039	798.3741	399.6907	797.3900	399.1987	9
8	723.2944	362.1508	705.2838	353.1456	S	758.3791	379.6932	741.3526	371.1799	740.3686	370.6879	8
9	820.3472	410.6772	802.3366	401.6719	P	671.3471	336.1772	654.3206	327.6639	653.3365	327.1719	7
10	877.3686	439.1880	859.3581	430.1827	G	574.2944	287.6508	557.2678	279.1375	556.2838	278.6455	6
11	978.4163	489.7118	960.4058	480.7065	T	517.2729	259.1401	500.2463	250.6268	499.2623	250.1348	5
12	1075.4691	538.2382	1057.4585	529.2329	P	416.2252	208.6162	399.1987	200.1030	398.2146	199.6110	4
13	1132.4905	566.7489	1114.4800	557.7436	G	319.1724	160.0899	302.1459	151.5766	301.1619	151.0846	3
14	1219.5226	610.2649	1201.5120	601.2596	S	262.1510	131.5791	245.1244	123.0659	244.1404	122.5738	2
15					R	175.1190	88.0631	158.0924	79.5498			1

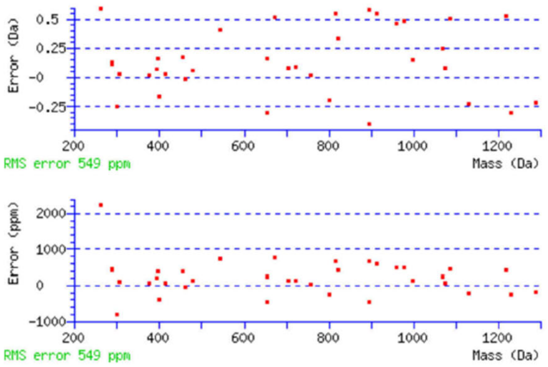

Figure S4: Annotated MS/MS spectra of phosphorylated Tau-X species derived from subcellular fractionation in fraction S1 (see Table S2) by Orbitrap LC-MS and PEAKS software analysis.

S4.A: peptide SGYSSPGS(+79.97)PGTPGSR, mass 1472.5933

Annotated spectrum with alignment

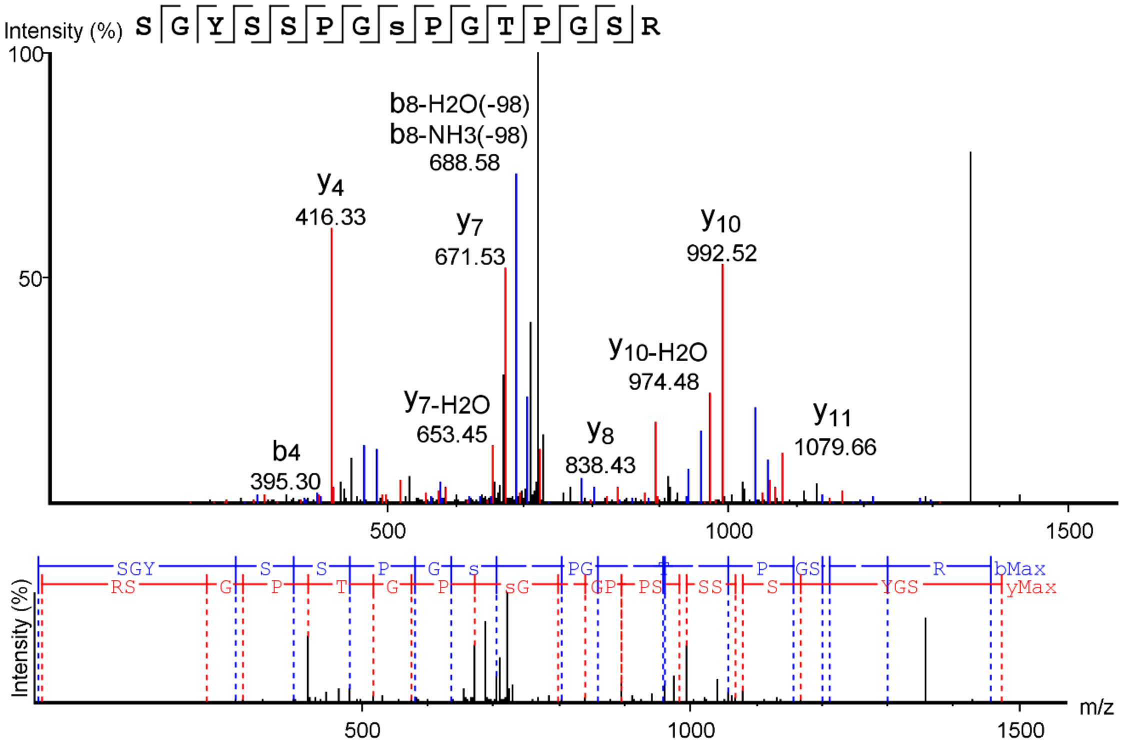

Ion table and mass error map

#	b	b-H2O	b-NH3	b (2+)	Seq	y	y-H2O	y-NH3	y (2+)	#
1	88.04	70.03	71.01	44.52	S					15
2	145.06	127.05	128.03	73.03	G	1386.57	1368.56	1369.54	693.20	14
3	308.09	289.67	291.10	154.56	Y	1329.55	1311.41	1312.52	665.27	13
4	395.30	377.23	378.45	198.08	S	1166.54	1148.47	1150.00	583.87	12
5	482.29	464.36	465.16	241.59	S	1079.66	1061.58	1062.42	540.23	11
6	579.36	561.25	562.24	289.67	P	992.52	974.48	975.39	496.89	10
7	636.36	618.63	619.37	318.72	G	895.11	877.63	878.34	448.18	9
8	802.96	785.38	786.23	402.13	S(+79.97)	838.43	820.73	820.73	419.32	8
9	900.31	882.65	883.43	450.66	P	671.53	653.45	654.32	335.58	7
10	957.30	939.21	940.51	479.17	G	574.45	556.62	557.27	287.65	6
11	1058.42	1040.47	1041.36	529.69	T	517.23	499.26	500.25	259.65	5
12	1155.60	1137.53	1138.41	577.65	P	416.33	398.30	399.51	209.07	4
13	1212.58	1194.45	1195.12	606.73	G	319.45	301.21	302.15	160.09	3
14	1299.64	1281.17	1282.62	650.41	S	262.18	244.09	245.12	131.58	2
15					R	175.12	157.11	158.09	88.06	1

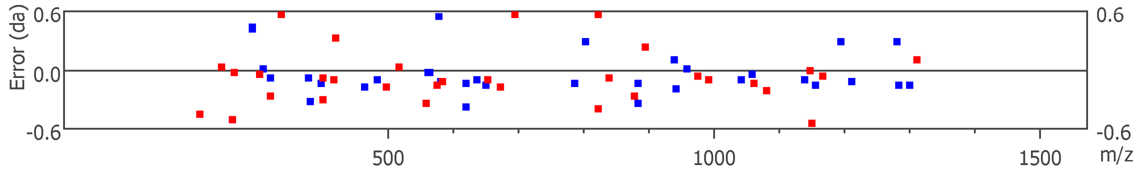

S4.B: peptide TPPAPKT(+79.97)PPSSGEPPK, mass 1666.7966

Annotated spectrum with alignment

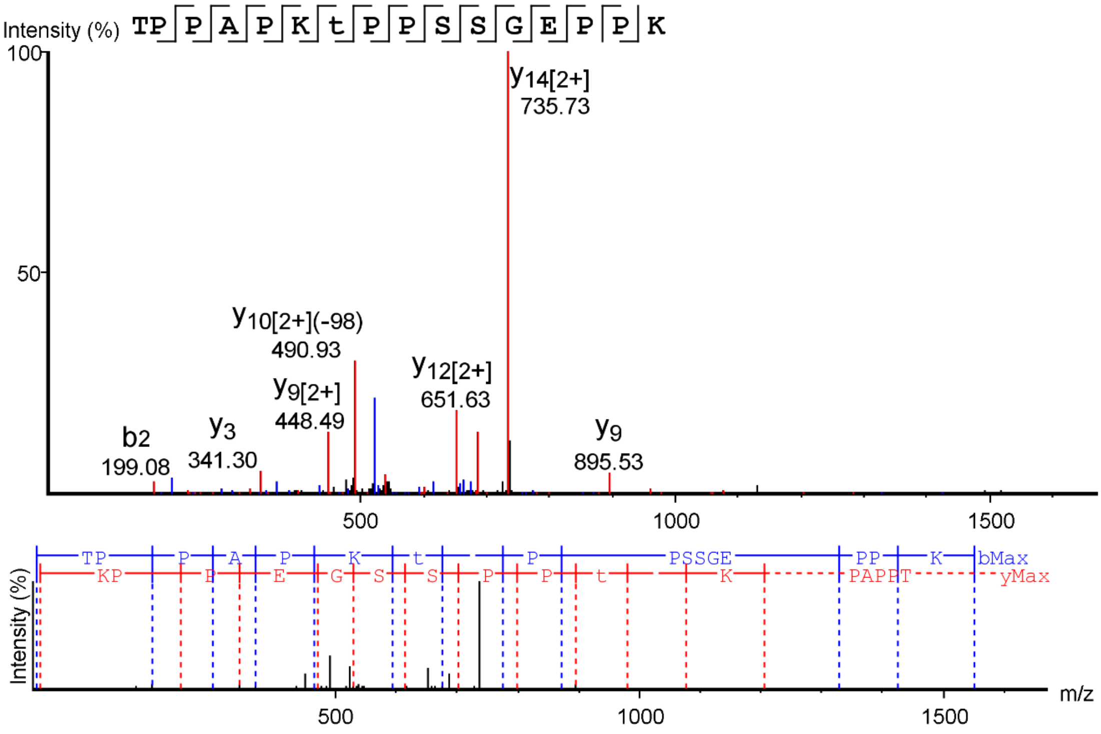

Ion table and mass error map

#	b	b-H2O	b-NH3	b (2+)	Seq	y	y-H2O	y-NH3	y (2+)	#
1	102.06	84.04	85.03	51.53	T					20
2	199.03	181.10	182.08	100.05	P	1981.94	1963.93	1964.91	991.47	19
3	296.25	278.30	279.13	148.58	P	1884.89	1866.87	1867.86	943.00	18
4	367.34	349.22	350.17	184.10	A	1787.83	1769.82	1770.81	894.30	17
5	464.25	446.24	447.22	232.63	P	1716.80	1698.78	1699.77	859.27	16
6	591.92	574.17	575.32	296.25	K	1619.74	1601.73	1602.72	810.81	15
7	773.44	755.35	756.45	387.18	T(+79.97)	1491.83	1473.64	1474.62	746.32	14
8	870.41	852.40	853.39	435.51	P	1310.72	1292.99	1293.61	655.82	13
9	967.47	949.45	950.44	484.23	P	1213.70	1195.57	1196.55	607.76	12
10	1054.50	1036.49	1037.47	528.08	S	1117.05	1098.52	1099.50	558.76	11
11	1141.53	1123.52	1124.50	571.41	S	1029.64	1011.49	1012.47	515.56	10
12	1198.55	1180.49	1181.52	599.37	G	943.00	924.82	925.44	471.73	9
13	1327.76	1309.73	1310.72	664.30	E	885.51	867.27	868.42	443.22	8
14	1424.65	1406.64	1407.62	712.82	P	756.45	738.39	739.59	378.99	7
15	1521.70	1503.69	1504.67	761.35	P	659.59	641.85	641.85	330.62	6
16	1649.79	1631.78	1632.77	825.40	K	562.52	544.28	545.27	281.27	5
17	1736.83	1718.82	1719.80	868.91	S	434.51	416.57	416.57	217.60	4
18	1793.85	1775.84	1776.82	897.11	G	347.17	329.16	330.62	174.08	3
19	1908.87	1890.86	1891.85	955.39	D	290.15	272.14	273.12	145.57	2
20					R	175.12	157.11	158.09	88.06	1

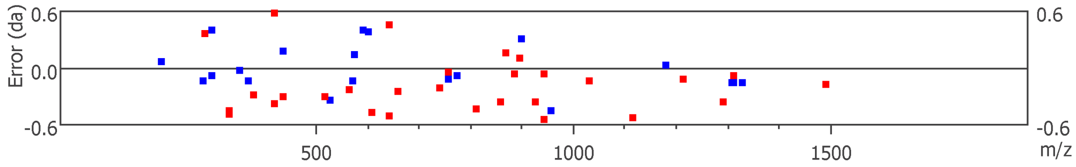

S4.C: peptide TPPAPKT(+79.97)PPSSGEPPKSGDR, mass 2081.9783

Annotated spectrum with alignment

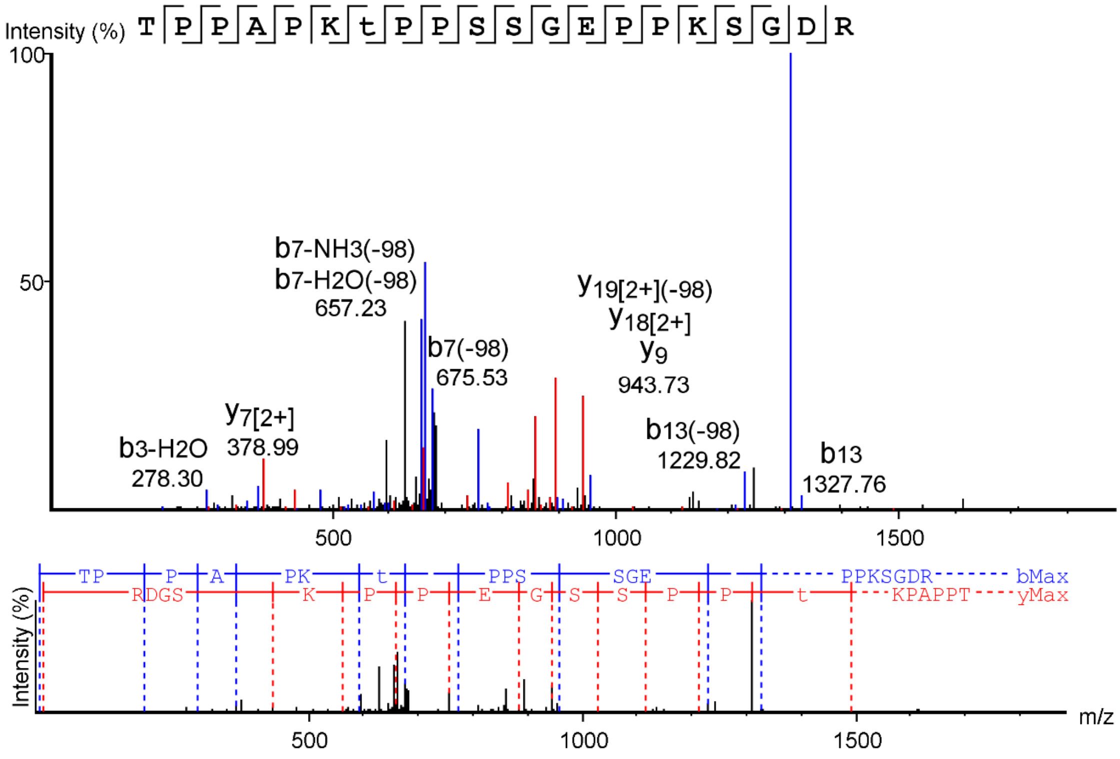

Ion table and mass error map

#	b	b-H2O	b-NH3	b (2+)	Seq	y	y-H2O	y-NH3	y (2+)	#
1	102.06	84.04	85.03	51.53	T					16
2	199.08	181.13	182.24	100.05	P	1566.76	1548.75	1549.73	783.88	15
3	296.37	278.24	279.13	148.58	P	1469.70	1451.69	1452.68	735.73	14
4	367.30	349.18	350.17	184.10	A	1372.65	1354.64	1355.62	686.82	13
5	464.29	446.24	447.22	232.19	P	1301.61	1283.89	1284.59	651.63	12
6	592.67	574.58	575.31	296.37	K	1204.25	1186.55	1187.53	602.67	11
7	773.40	755.35	756.03	387.18	T(+79.97)	1076.58	1058.33	1059.11	538.43	10
8	870.64	852.45	853.39	435.71	P	895.53	877.54	878.43	448.49	9
9	967.47	949.45	950.44	484.23	P	798.73	780.77	781.37	399.59	8
10	1054.50	1036.49	1037.47	527.68	S	701.64	683.34	684.07	351.23	7
11	1141.53	1123.52	1124.50	570.80	S	614.28	596.27	597.64	307.78	6
12	1198.55	1180.54	1181.52	599.78	G	527.68	509.68	509.68	264.27	5
13	1327.86	1309.58	1310.57	664.59	E	470.48	452.52	453.23	235.40	4
14	1424.65	1406.64	1407.62	712.82	P	341.30	323.24	324.57	171.05	3
15	1521.70	1503.69	1504.67	761.29	P	244.22	226.23	226.95	122.58	2
16					K	147.11	129.10	130.09	74.06	1

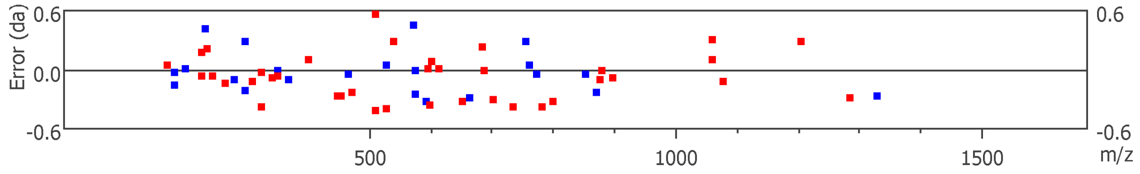

S4.D: peptide VAVVRT(+79.97)PPKSPSSAK, mass 1602.8494

Annotated spectrum with alignment

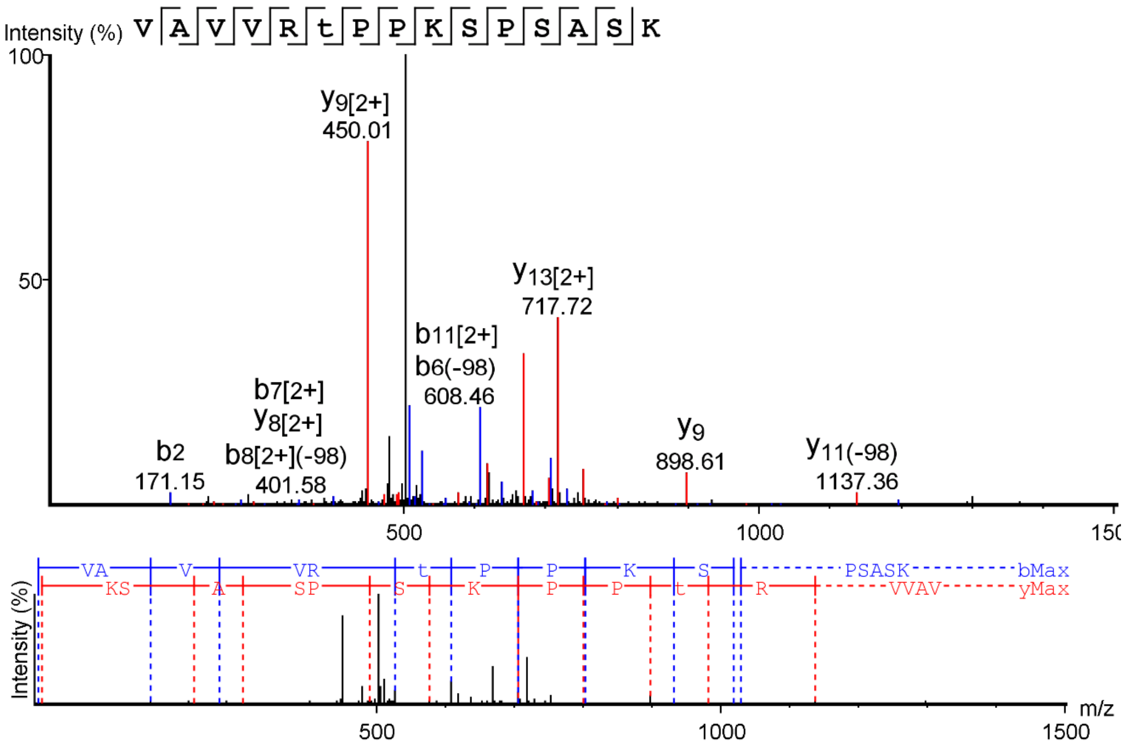

Ion table and mass error map

#	b	b-H2O	b-NH3	b (2+)	Seq	y	y-H2O	y-NH3	y (2+)	#
1	100.08	82.07	83.05	50.54	V					15
2	171.15	153.10	154.09	86.06	A	1504.79	1486.78	1487.76	753.15	14
3	270.23	252.17	253.15	135.59	V	1433.75	1415.74	1416.72	717.72	13
4	369.25	350.87	351.77	185.13	V	1334.68	1316.67	1317.66	668.31	12
5	525.33	507.02	508.79	262.64	R	1235.61	1217.60	1218.59	618.78	11
6	706.46	688.27	689.57	353.42	T(+79.97)	1079.51	1061.50	1062.49	539.97	10
7	803.54	785.14	786.32	402.27	P	898.61	880.49	881.28	450.01	9
8	900.47	882.69	883.59	450.74	P	801.39	783.32	784.42	401.58	8
9	1028.93	1010.56	1011.54	514.67	K	704.24	686.82	686.82	352.70	7
10	1115.60	1097.59	1098.57	558.45	S	576.36	558.45	559.27	289.11	6
11	1212.65	1194.69	1195.62	606.34	P	489.46	471.26	472.24	245.38	5
12	1299.68	1281.67	1282.66	650.34	S	392.21	373.92	375.19	197.19	4
13	1370.72	1352.71	1353.69	685.86	A	305.24	287.17	288.15	153.09	3
14	1457.75	1439.74	1440.72	729.69	S	234.11	216.13	217.07	117.57	2
15					K	147.11	129.10	130.09	74.06	1

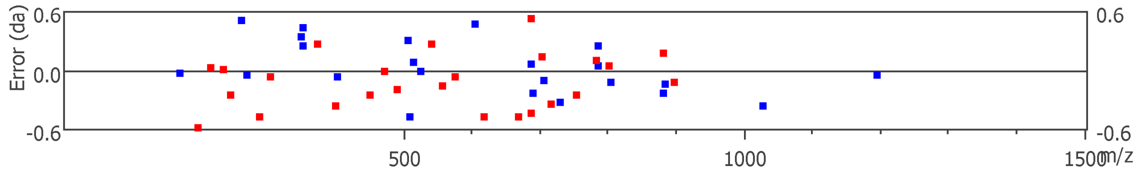

Figure S5: Annotated MS/MS spectra of phosphorylated Tau-X species derived from subcellular fractionation in fraction S2 (see Table S2) by Orbitrap LC-MS and PEAKS software analysis.

S5.A: peptide SGYSSPGS(+79.97)PGTPGSR, mass 1472.5933

Annotated spectrum with alignment

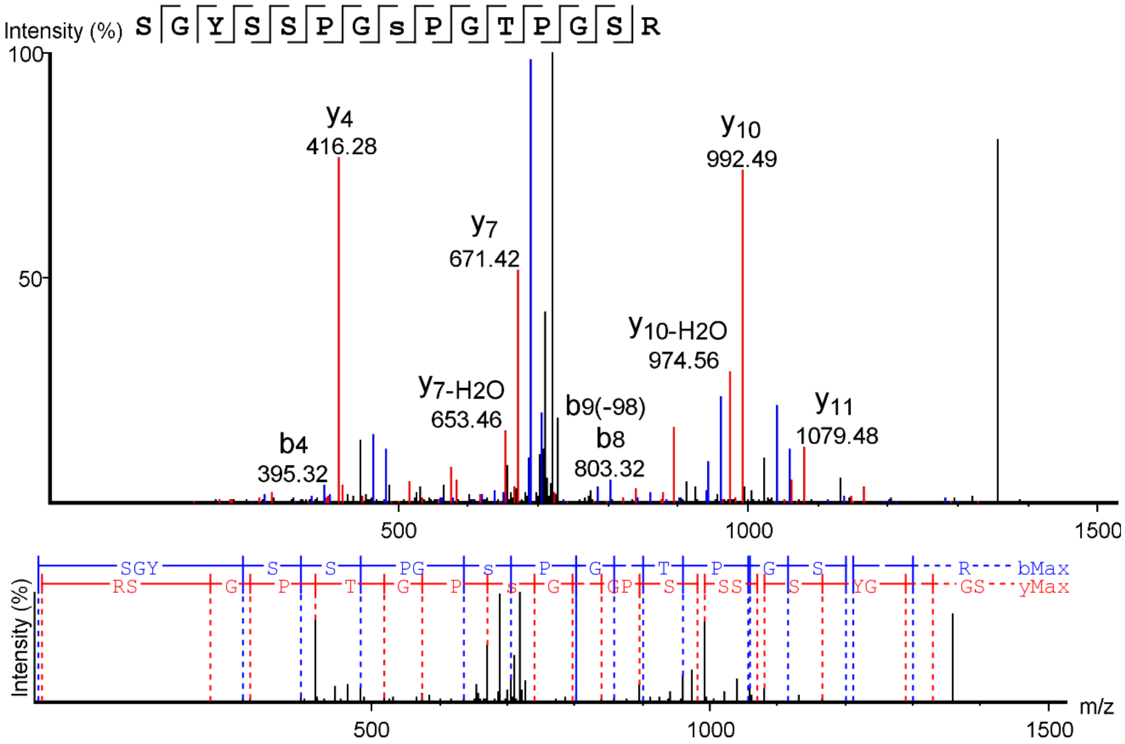

Ion table and mass error map

#	b	b-H2O	b-NH3	b (2+)	Seq	y	y-H2O	y-NH3	y (2+)	#
1	88.04	70.03	71.01	44.52	S					15
2	145.06	127.05	128.03	73.03	G	1386.57	1368.56	1369.54	693.78	14
3	308.21	290.21	291.37	154.56	Y	1329.45	1311.54	1312.52	665.37	13
4	395.32	377.20	378.50	198.08	S	1166.51	1148.61	1149.45	584.02	12
5	482.32	464.19	465.16	241.59	S	1079.48	1061.50	1062.42	540.23	11
6	579.24	561.34	562.47	290.21	P	992.49	974.56	975.39	496.71	10
7	636.82	618.45	619.24	318.63	G	895.37	877.58	878.34	448.40	9
8	803.32	785.29	786.23	402.48	S(+79.97)	838.42	820.30	821.53	419.28	8
9	900.62	882.04	883.51	450.66	P	671.42	653.46	654.32	336.17	7
10	957.34	939.41	940.31	478.64	G	574.51	556.28	557.13	287.65	6
11	1058.44	1040.34	1041.52	529.69	T	517.40	499.34	500.79	259.27	5
12	1155.44	1137.29	1138.23	578.53	P	416.28	398.32	399.43	209.11	4
13	1212.65	1194.60	1195.43	606.28	G	319.30	300.98	302.22	160.09	3
14	1299.81	1281.48	1282.12	650.06	S	262.26	244.20	245.24	131.58	2
15					R	175.12	157.11	158.09	88.06	1

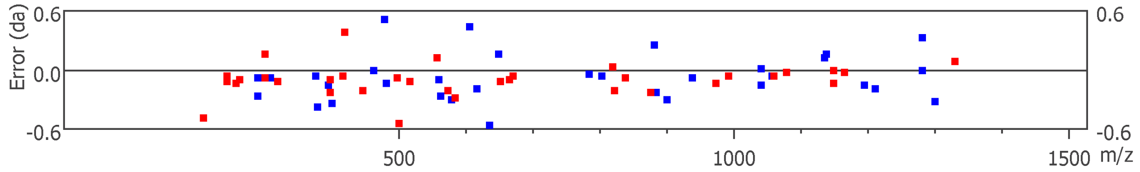

5.B: peptide SGYSS(+79.97)PGSPGTPGSR, mass 1472.5933

Annotated spectrum with alignment

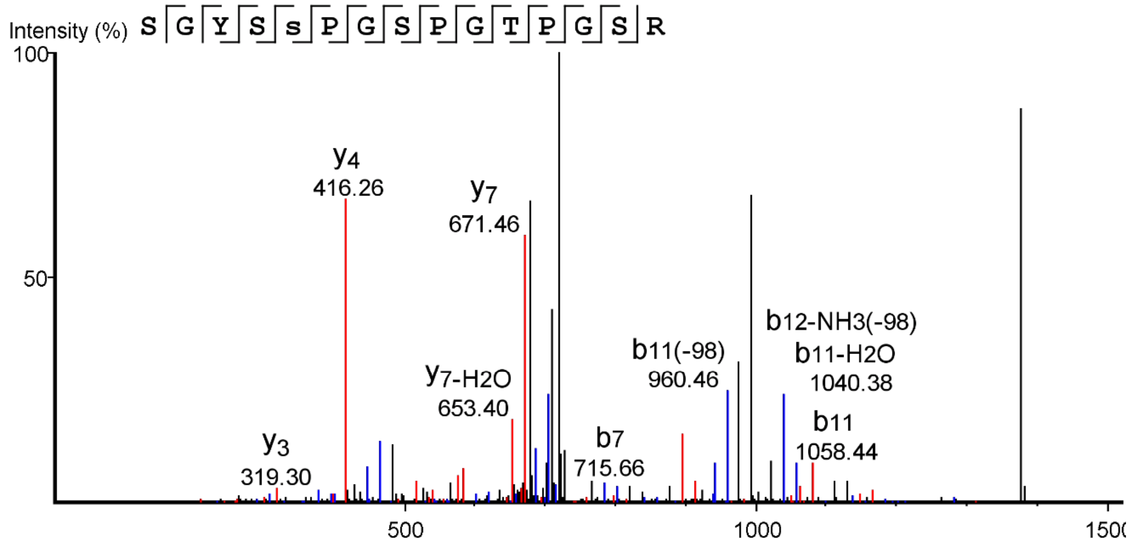

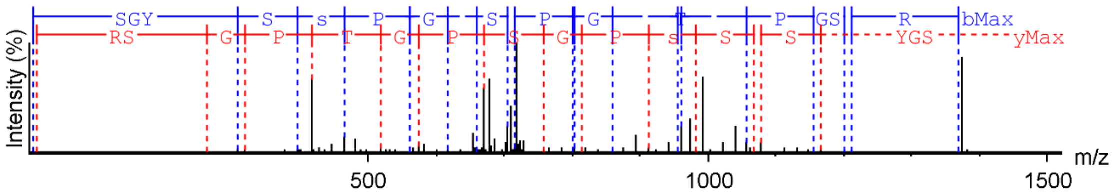

Ion table and mass error map

#	b	b-H2O	b-NH3	b (2+)	Seq	y	y-H2O	y-NH3	y (2+)	#
1	88.04	70.03	71.01	44.52	S					15
2	145.06	127.05	128.03	73.03	G	1386.57	1368.56	1369.54	694.10	14
3	308.21	290.25	291.10	154.56	Y	1329.55	1311.54	1312.98	665.40	13
4	395.20	377.24	378.13	198.08	S	1166.62	1148.60	1149.46	583.86	12
5	562.48	544.14	545.20	281.58	S(+79.97)	1079.52	1061.53	1062.70	540.31	11
6	659.09	641.20	642.18	329.54	P	912.68	894.58	895.43	456.73	10
7	715.66	698.19	698.97	358.06	G	815.77	797.50	798.37	408.20	9
8	803.32	785.54	786.23	402.13	S	758.66	740.39	741.94	379.69	8
9	900.31	882.30	883.78	450.29	P	671.46	653.40	654.32	336.17	7
10	957.20	939.71	939.71	479.17	G	574.78	556.23	557.27	287.65	6
11	1058.44	1040.38	1041.36	529.15	T	517.41	499.26	500.25	259.19	5
12	1155.86	1137.71	1138.41	578.22	P	416.26	398.29	399.20	209.10	4
13	1212.82	1194.45	1195.05	606.73	G	319.30	301.19	302.15	160.09	3
14	1299.49	1281.46	1282.77	649.72	S	262.09	244.08	245.12	131.58	2
15					R	175.12	157.11	158.09	88.06	1

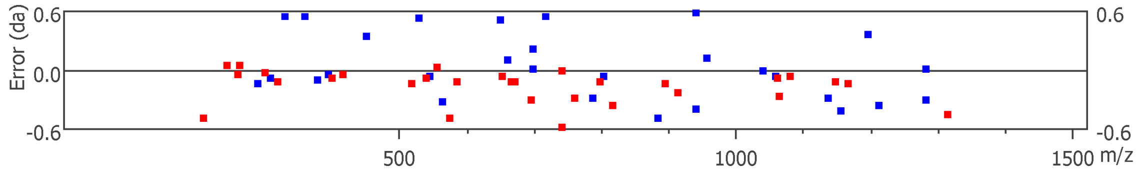

S5.C: peptide TPPAPKT(+79.97)PPSSGEPPK, mass 1666.7966

Annotated spectrum with alignment

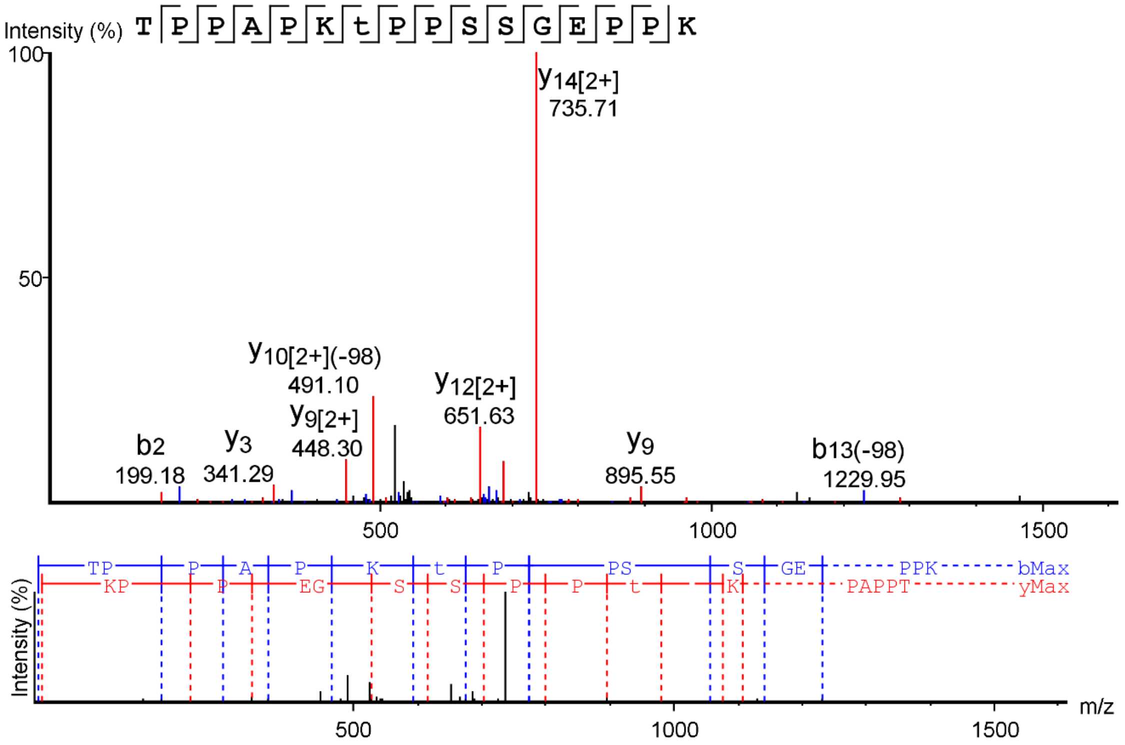

Ion table and mass error map

#	b	b-H2O	b-NH3	b (2+)	Seq	y	y-H2O	y-NH3	y (2+)	#
1	102.06	84.04	85.03	51.53	T					16
2	199.18	181.10	182.08	100.05	P	1566.76	1548.75	1549.73	784.28	15
3	296.26	278.22	279.13	148.58	P	1469.70	1451.69	1452.68	735.71	14
4	367.35	349.28	350.17	184.10	A	1372.65	1354.64	1355.62	686.85	13
5	463.88	446.24	447.22	232.63	P	1301.61	1283.82	1284.59	651.63	12
6	592.37	574.34	575.32	296.26	K	1204.56	1186.55	1187.74	602.40	11
7	773.49	755.60	756.91	387.21	T(+79.97)	1076.64	1058.84	1059.83	538.73	10
8	870.41	851.82	853.39	435.71	P	895.55	877.58	878.39	448.30	9
9	967.47	949.45	950.44	484.67	P	798.61	780.39	781.58	399.70	8
10	1054.67	1036.49	1037.47	527.67	S	701.36	683.33	684.32	351.17	7
11	1141.52	1123.52	1124.50	571.26	S	614.46	596.50	597.86	308.19	6
12	1198.55	1180.54	1181.52	599.58	G	527.67	509.55	510.26	264.42	5
13	1327.59	1309.58	1310.57	664.59	E	470.26	452.45	453.23	235.63	4
14	1424.65	1406.64	1407.62	712.44	P	341.29	323.36	324.19	171.19	3
15	1521.70	1503.69	1504.67	761.35	P	244.33	226.19	227.14	122.58	2
16					K	147.11	129.10	130.09	74.06	1

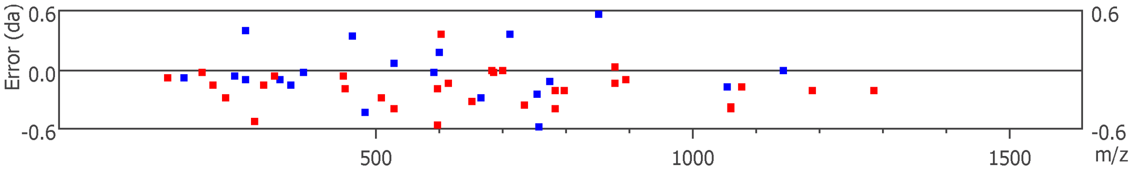

S5.D: peptide TPPAPKT(+79.97)PPSSGEPPKSGDR, mass 2081.9783

Annotated spectrum with alignment

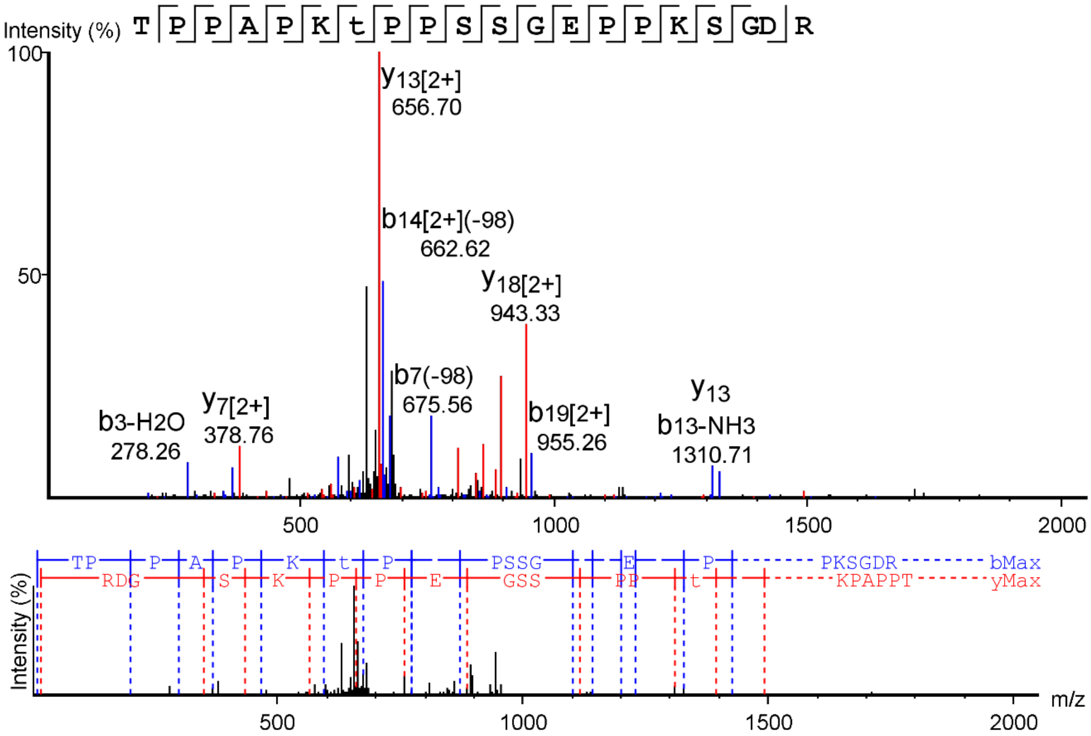

Ion table and mass error map

#	b	b-H2O	b-NH3	b (2+)	Seq	y	y-H2O	y-NH3	y (2+)	#
1	102.06	84.04	85.03	51.53	T					20
2	199.28	181.10	182.08	100.05	P	1981.94	1963.93	1964.91	991.90	19
3	296.08	278.26	279.13	148.58	P	1884.89	1866.87	1867.86	943.33	18
4	367.35	349.17	350.44	184.10	A	1787.83	1769.82	1770.81	894.41	17
5	464.44	446.24	447.32	233.14	P	1716.80	1698.78	1699.77	859.01	16
6	592.47	574.38	575.32	296.08	K	1619.74	1601.73	1602.72	810.75	15
7	773.47	755.43	756.53	387.18	T(+79.97)	1491.72	1473.64	1474.62	746.54	14
8	870.80	852.40	853.39	435.71	P	1310.71	1292.37	1293.72	655.98	13
9	967.47	949.45	950.76	484.23	P	1213.58	1195.57	1196.55	606.76	12
10	1054.50	1035.97	1037.47	527.53	S	1116.68	1098.77	1099.50	558.94	11
11	1141.85	1123.06	1124.50	571.26	S	1029.50	1011.49	1012.47	514.66	10
12	1198.55	1180.58	1181.52	600.13	G	942.46	924.02	925.61	471.86	9
13	1327.71	1309.17	1310.71	664.13	E	885.34	867.71	868.51	443.22	8
14	1424.51	1406.64	1407.62	712.82	P	756.53	738.39	739.57	378.76	7
15	1521.70	1503.69	1505.21	760.77	P	659.54	641.63	642.43	330.55	6
16	1649.79	1631.78	1632.97	825.23	K	562.49	543.74	545.72	281.65	5
17	1736.83	1718.82	1719.80	868.51	S	434.42	416.43	417.17	218.17	4
18	1793.85	1775.84	1776.82	897.42	G	347.50	329.16	330.55	174.08	3
19	1908.87	1890.86	1891.85	955.26	D	290.15	272.14	273.12	145.57	2
20					R	175.12	157.11	158.09	88.06	1

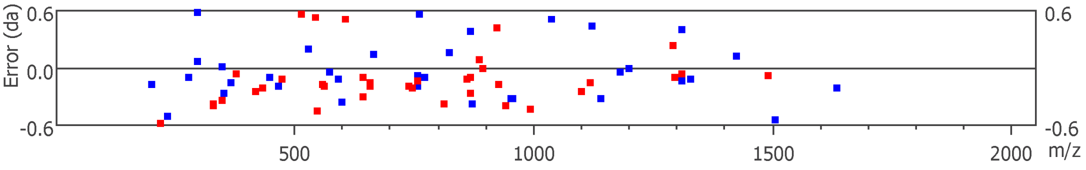

S5.E: peptide VAVVRT(+79.97)PPKSPSSAK, mass 1602.8494

Annotated spectrum with alignment

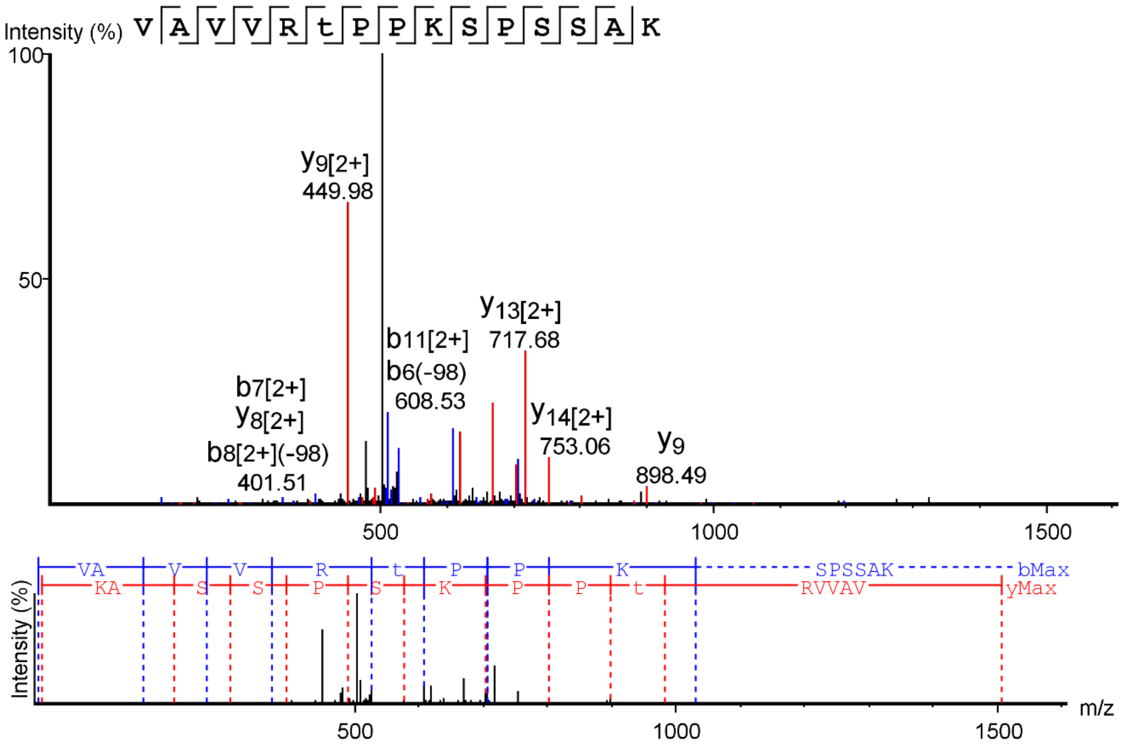

Ion table and mass error map

#	b	b-H2O	b-NH3	b (2+)	Seq	y	y-H2O	y-NH3	y (2+)	#
1	100.08	82.07	83.05	50.54	V					15
2	171.00	153.10	154.09	86.06	A	1504.79	1486.78	1487.76	753.06	14
3	270.24	252.34	253.15	135.59	V	1433.75	1415.74	1416.72	717.68	13
4	369.33	351.24	352.22	185.13	V	1334.68	1316.67	1317.66	668.37	12
5	525.56	507.05	508.22	263.18	R	1235.61	1217.60	1218.59	618.31	11
6	706.49	688.54	689.85	353.67	T(+79.97)	1079.51	1061.43	1062.49	540.53	10
7	803.42	785.41	786.08	402.41	P	898.49	880.46	881.58	449.98	9
8	900.47	882.55	883.92	450.74	P	801.43	783.76	784.42	401.51	8
9	1029.12	1010.56	1011.54	514.79	K	704.22	686.43	687.37	352.88	7
10	1115.60	1097.59	1098.57	558.34	S	576.39	558.34	559.27	288.90	6
11	1212.65	1194.64	1195.40	607.39	P	489.29	471.34	472.24	245.13	5
12	1299.68	1281.67	1282.66	649.99	S	392.13	374.20	375.19	197.02	4
13	1386.71	1368.70	1369.69	693.86	S	304.77	287.17	288.14	153.09	3
14	1457.75	1439.74	1440.72	729.62	A	218.53	200.34	201.12	109.57	2
15					K	147.11	129.10	130.09	74.06	1

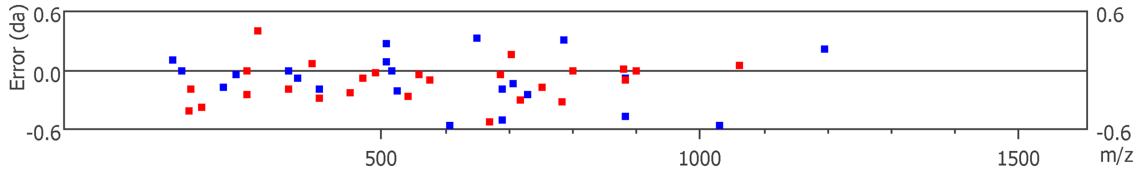

S5.F: peptide KVAVVRT(+79.97)PPKSPSSAK, mass 1730.9443

Annotated spectrum with alignment

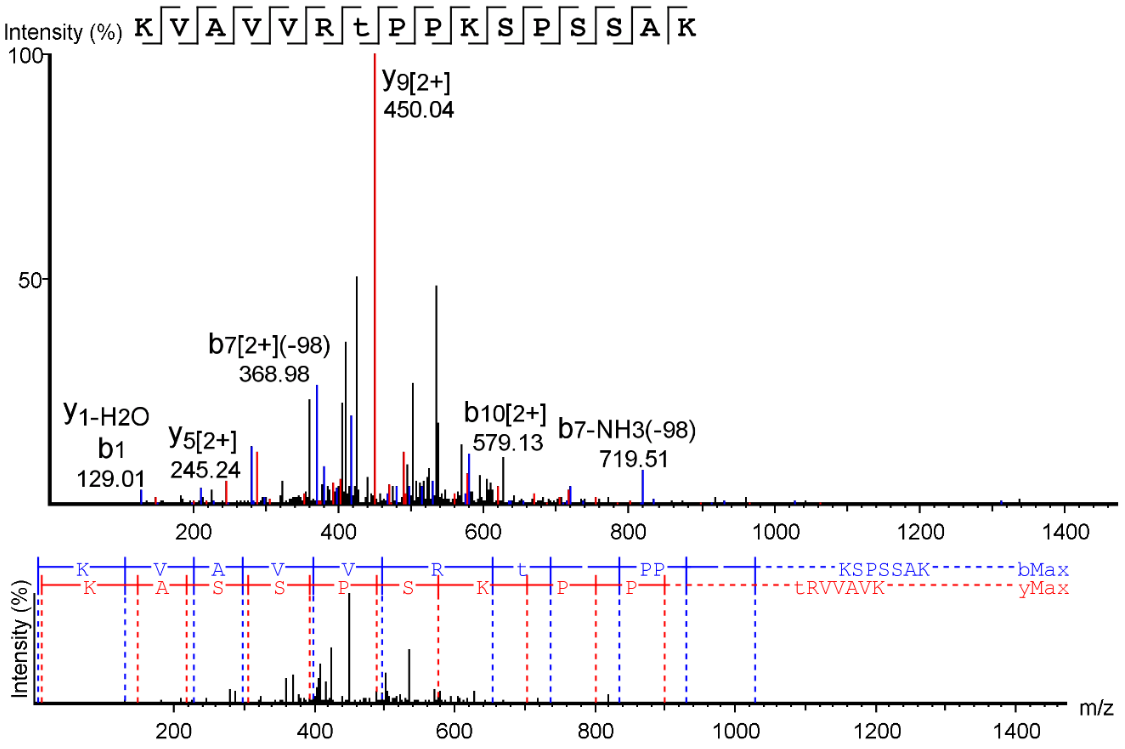

Ion table and mass error map

#	b	b-H2O	b-NH3	b (2+)	Seq	y	y-H2O	y-NH3	y (2+)	#
1	129.01	111.09	112.08	65.05	K					16
2	228.32	210.15	211.21	114.59	V	1603.86	1585.85	1586.83	802.43	15
3	299.03	280.89	282.16	150.17	A	1504.79	1486.78	1487.76	752.94	14
4	398.61	380.52	381.34	199.24	V	1433.75	1415.74	1416.72	717.70	13
5	496.90	479.51	480.48	249.04	V	1334.68	1316.67	1317.66	668.27	12
6	653.37	635.26	636.53	327.22	R	1235.61	1217.60	1218.59	618.66	11
7	834.65	816.40	817.67	417.95	T(+79.97)	1079.51	1061.50	1062.88	540.26	10
8	931.23	913.50	914.75	466.36	P	898.09	880.49	881.47	450.04	9
9	1028.83	1010.56	1011.54	514.93	P	801.54	782.86	784.42	401.52	8
10	1156.66	1138.65	1139.63	579.13	K	704.03	686.40	687.57	352.84	7
11	1243.69	1225.68	1226.67	622.35	S	576.40	558.58	559.27	288.92	6
12	1340.75	1322.73	1323.72	670.39	P	489.34	471.04	471.77	245.24	5
13	1427.78	1409.77	1410.75	714.93	S	392.37	373.75	375.48	197.14	4
14	1514.81	1496.80	1497.78	757.90	S	305.34	287.17	288.15	153.09	3
15	1585.85	1567.84	1568.82	793.42	A	218.12	200.25	201.17	109.57	2
16					K	147.12	129.01	130.09	74.06	1

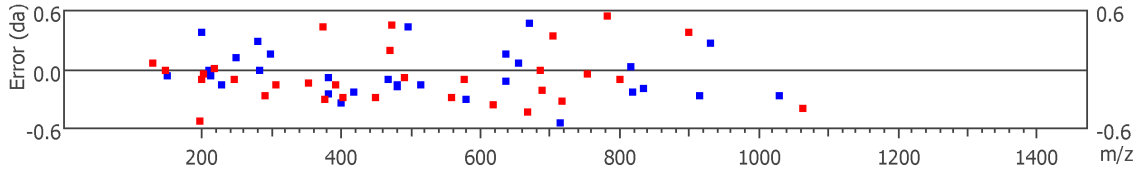

Figure S6: Annotated MS/MS spectra of phosphorylated Tau-X species derived from subcellular fractionation in fraction S3 (see Table S2) by Orbitrap LC-MS and PEAKS software analysis.

S6.A: peptide TPPAPKT(+79.97)PPSSGEPPK, mass 1666.7966

Annotated spectrum with alignment

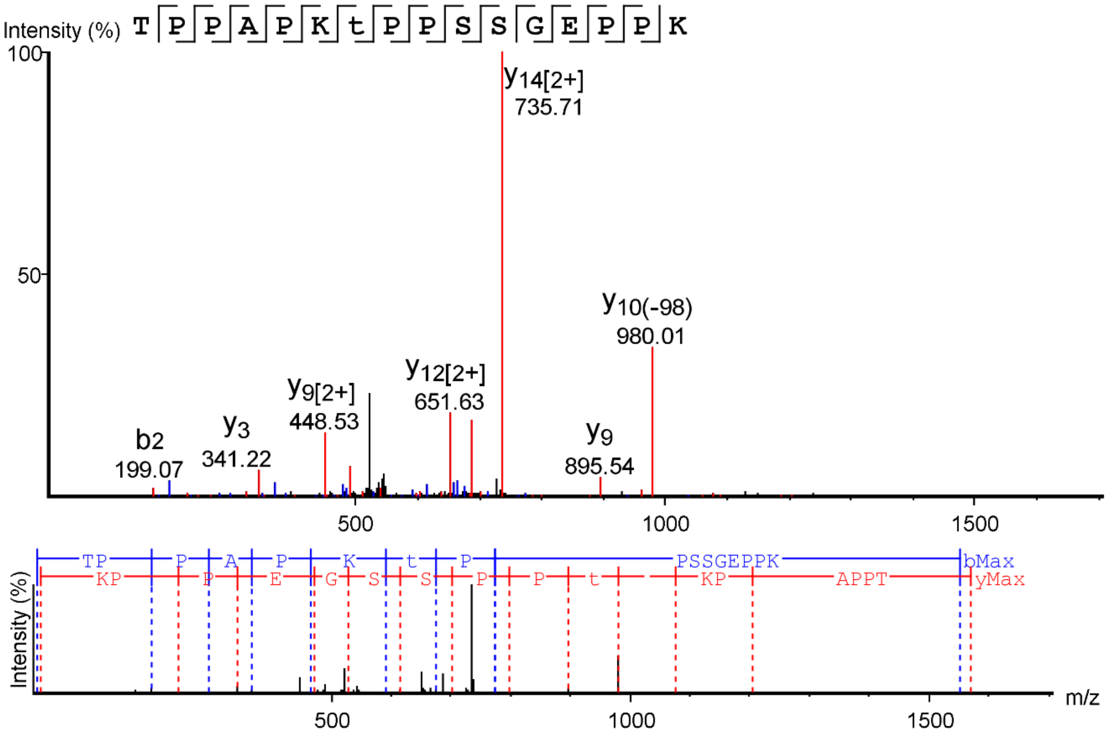

Ion table and mass error map

#	b	b-H2O	b-NH3	b (2+)	Seq	y	y-H2O	y-NH3	y (2+)	#
1	102.06	84.04	85.03	51.53	T					16
2	199.07	181.10	182.10	100.05	P	1566.76	1548.75	1549.73	783.78	15
3	296.24	277.85	279.40	148.58	P	1469.70	1451.69	1452.68	735.71	14
4	367.27	349.13	350.33	184.10	A	1372.65	1354.64	1355.62	686.82	13
5	464.21	445.96	447.22	232.33	P	1301.61	1283.60	1284.59	651.63	12
6	592.44	573.96	575.32	296.24	K	1204.56	1186.55	1187.52	602.87	11
7	773.45	755.35	756.33	387.07	T(+79.97)	1076.56	1058.85	1058.85	538.53	10
8	870.41	852.40	853.39	435.49	P	895.54	877.70	878.42	448.53	9
9	967.47	949.45	950.44	483.79	P	798.46	780.39	781.37	400.25	8
10	1054.50	1036.68	1037.47	527.61	S	701.33	683.31	684.59	351.22	7
11	1141.53	1123.52	1124.50	571.26	S	614.28	596.58	597.29	307.66	6
12	1198.55	1180.54	1181.52	599.84	G	527.61	509.79	509.79	264.35	5
13	1327.59	1309.58	1310.57	664.49	E	470.25	452.42	453.13	235.63	4
14	1424.65	1406.64	1407.62	712.24	P	341.22	323.42	324.19	171.18	3
15	1521.70	1503.69	1504.67	761.82	P	244.31	226.20	227.14	122.58	2
16					K	147.11	129.10	130.09	74.06	1

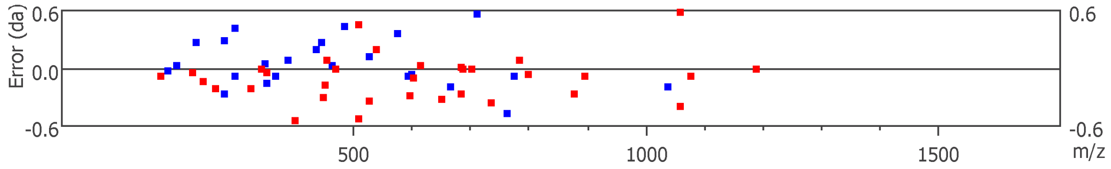

S6.B: peptide TPPAPKT(+79.97)PPSSGEPPKSGDR, mass 2081.9783

Annotated spectrum with alignment

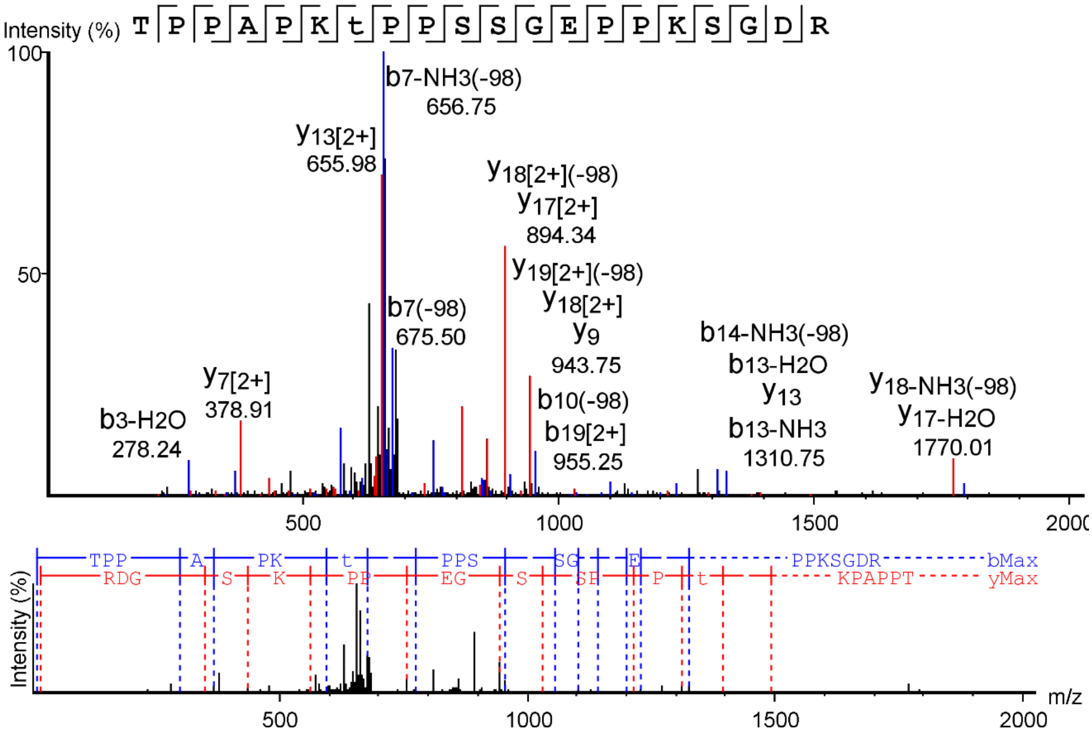

Ion table and mass error map

#	b	b-H2O	b-NH3	b (2+)	Seq	y	y-H2O	y-NH3	y (2+)	#
1	102.06	84.04	85.03	51.53	T					20
2	199.11	181.10	182.08	100.05	P	1981.94	1963.93	1964.91	991.47	19
3	296.32	278.24	279.13	148.58	P	1884.89	1866.87	1867.86	942.93	18
4	367.20	349.15	350.17	184.10	A	1787.83	1770.01	1770.81	894.34	17
5	464.25	446.24	447.22	232.18	P	1716.80	1698.78	1699.77	859.32	16
6	592.39	574.34	575.12	296.32	K	1619.74	1601.73	1602.72	810.23	15
7	773.47	754.96	756.48	387.18	T(+79.97)	1491.42	1473.64	1474.62	746.32	14
8	870.41	852.40	853.51	435.43	P	1310.75	1292.88	1293.61	655.98	13
9	967.47	949.45	950.44	484.26	P	1213.51	1195.57	1196.55	607.78	12
10	1054.85	1036.51	1037.47	527.35	S	1116.53	1098.52	1099.50	558.74	11
11	1141.56	1123.52	1124.50	571.26	S	1029.79	1011.49	1012.47	515.31	10
12	1198.76	1180.54	1181.52	599.78	G	942.93	924.45	925.41	472.03	9
13	1327.75	1309.73	1310.75	664.61	E	885.44	867.60	868.39	443.34	8
14	1424.65	1406.64	1407.62	712.82	P	756.48	738.22	739.80	378.91	7
15	1521.70	1503.69	1504.67	761.35	P	659.35	641.07	642.58	330.30	6
16	1649.79	1631.78	1632.77	825.40	K	562.49	544.28	545.00	281.99	5
17	1736.83	1718.82	1719.80	868.39	S	434.20	416.36	417.17	218.07	4
18	1793.85	1775.84	1776.82	897.42	G	347.27	329.16	330.30	174.08	3
19	1908.87	1890.86	1891.85	955.25	D	290.15	272.14	273.12	145.57	2
20					R	175.12	157.11	158.09	88.06	1

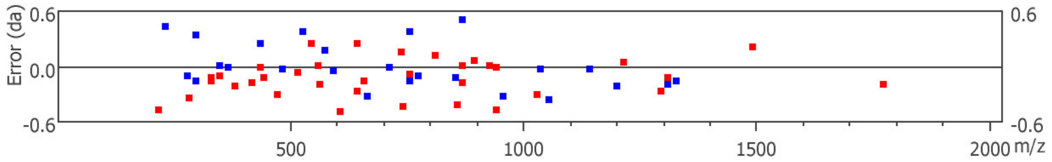

S6.C: peptide VAVVRT(+79.97)PPKSPSSAK, mass 1602.8494

Annotated spectrum with alignment

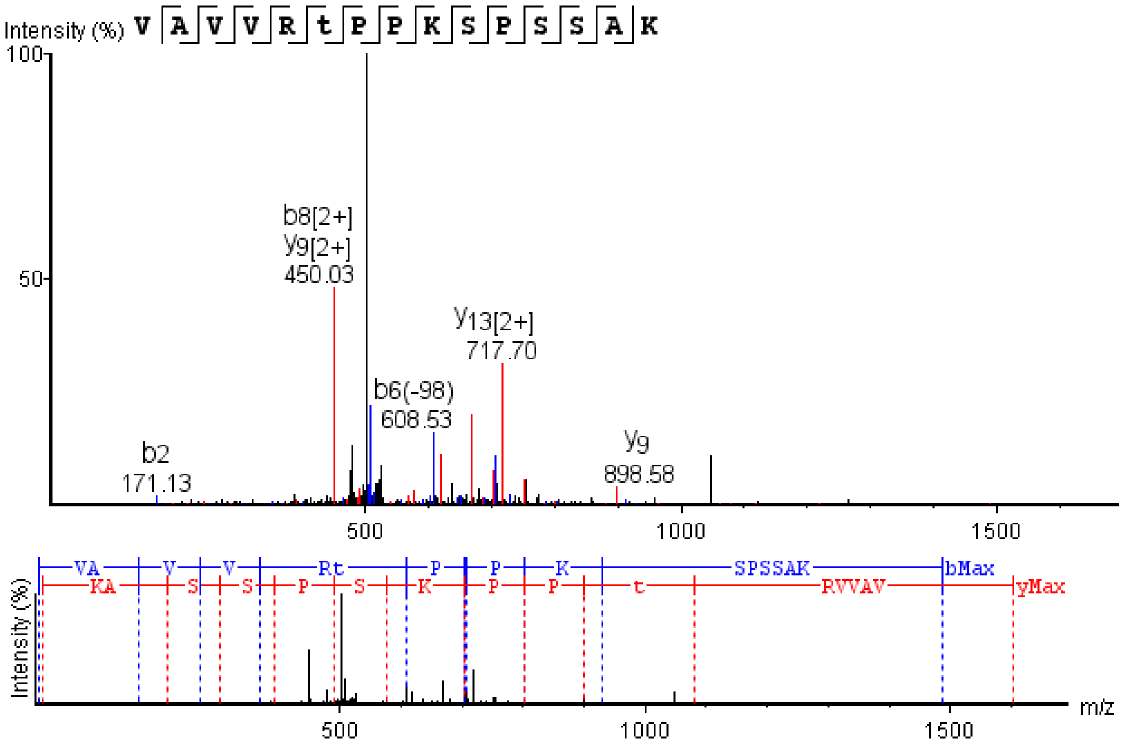

Ion table and mass error map

#	b	b-H2O	b-NH3	b (2+)	Seq	y	y-H2O	y-NH3	y (2+)	#
1	100.08	82.07	83.05	50.54	V					15
2	171.13	153.10	154.09	86.06	A	1504.79	1487.06	1487.76	752.91	14
3	270.07	252.32	253.15	135.59	V	1433.75	1415.74	1416.72	717.70	13
4	369.12	351.28	352.73	185.13	V	1334.68	1316.67	1317.66	668.38	12
5	525.35	506.76	508.04	263.18	R	1235.61	1217.60	1219.03	618.31	11
6	706.45	688.32	689.52	353.59	T(+79.97)	1079.67	1061.50	1062.49	540.50	10
7	803.42	785.78	786.62	402.21	P	898.58	880.72	881.47	450.03	9
8	900.47	882.46	883.44	451.07	P	801.51	783.58	784.42	401.53	8
9	1028.57	1010.56	1011.54	514.67	K	704.28	686.47	687.60	352.73	7
10	1115.60	1097.59	1098.57	558.46	S	576.48	558.46	559.27	288.73	6
11	1212.65	1194.64	1195.62	606.83	P	489.38	471.20	472.15	245.43	5
12	1299.68	1281.67	1282.66	650.40	S	392.20	374.20	375.43	196.22	4
13	1386.71	1368.70	1369.69	693.86	S	305.26	287.17	288.73	153.09	3
14	1457.75	1439.74	1440.72	729.59	A	218.20	200.14	201.12	109.57	2
15					K	147.11	129.10	130.09	74.06	1

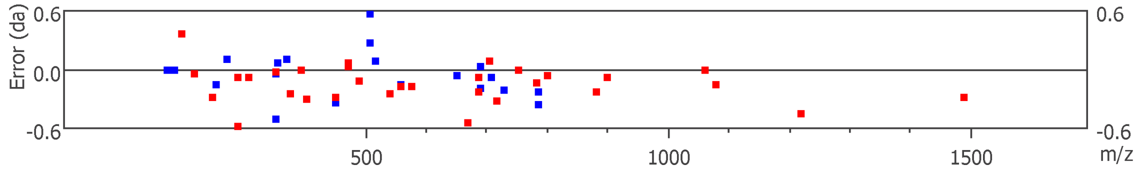

S6.D: peptide KVAVVRT(+79.97)PPKSPSSAK, mass 1730.9443

Annotated spectrum with alignment

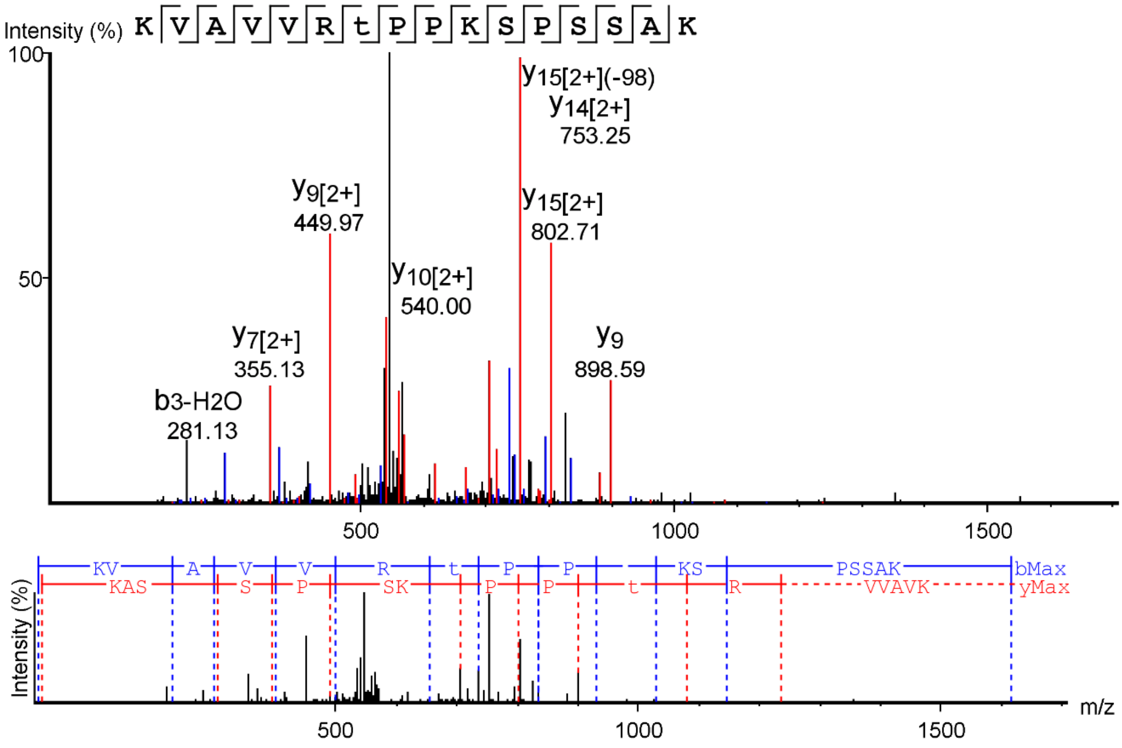

Ion table and mass error map

#	b	b-H2O	b-NH3	b (2+)	Seq	y	y-H2O	y-NH3	y (2+)	#
1	129.10	111.09	112.08	65.05	K					16
2	228.13	210.33	211.21	114.59	V	1603.86	1585.85	1586.83	802.71	15
3	299.30	281.13	282.18	150.10	A	1504.79	1486.78	1487.76	753.25	14
4	398.48	380.27	381.28	200.23	V	1433.75	1415.74	1416.72	717.26	13
5	497.36	479.33	480.01	248.96	V	1334.68	1316.67	1317.66	668.11	12
6	653.54	635.44	636.42	327.22	R	1235.89	1217.60	1218.59	618.78	11
7	834.54	816.45	817.56	418.05	T(+79.97)	1079.90	1062.08	1062.08	540.00	10
8	931.51	913.50	914.49	466.26	P	898.59	880.58	881.47	449.97	9
9	1028.58	1010.56	1011.85	514.78	P	801.39	783.48	784.49	401.26	8
10	1156.66	1138.65	1139.63	578.83	K	704.20	686.31	687.68	353.09	7
11	1243.69	1225.68	1226.67	622.03	S	576.30	558.52	559.49	288.65	6
12	1340.75	1322.73	1323.72	670.52	P	489.10	471.26	472.30	245.10	5
13	1427.78	1409.77	1410.75	714.39	S	392.37	374.46	375.21	197.16	4
14	1514.81	1496.80	1497.78	758.15	S	305.29	287.17	287.90	153.09	3
15	1585.85	1567.84	1568.82	793.60	A	218.15	200.23	201.24	109.57	2
16					K	147.11	129.10	130.09	74.06	1

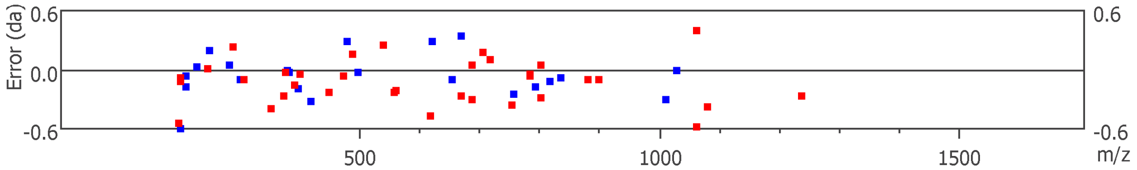

Figure S7: Annotated MS/MS spectra of phosphorylated Tau-X species derived from subcellular fractionation in fraction LP1 (see Table S2) by Orbitrap LC-MS and PEAKS software analysis.

S7.A: peptide SGYSSPGS(+79.97)PGTPGSR, mass 1472.5933

Annotated spectrum with alignment

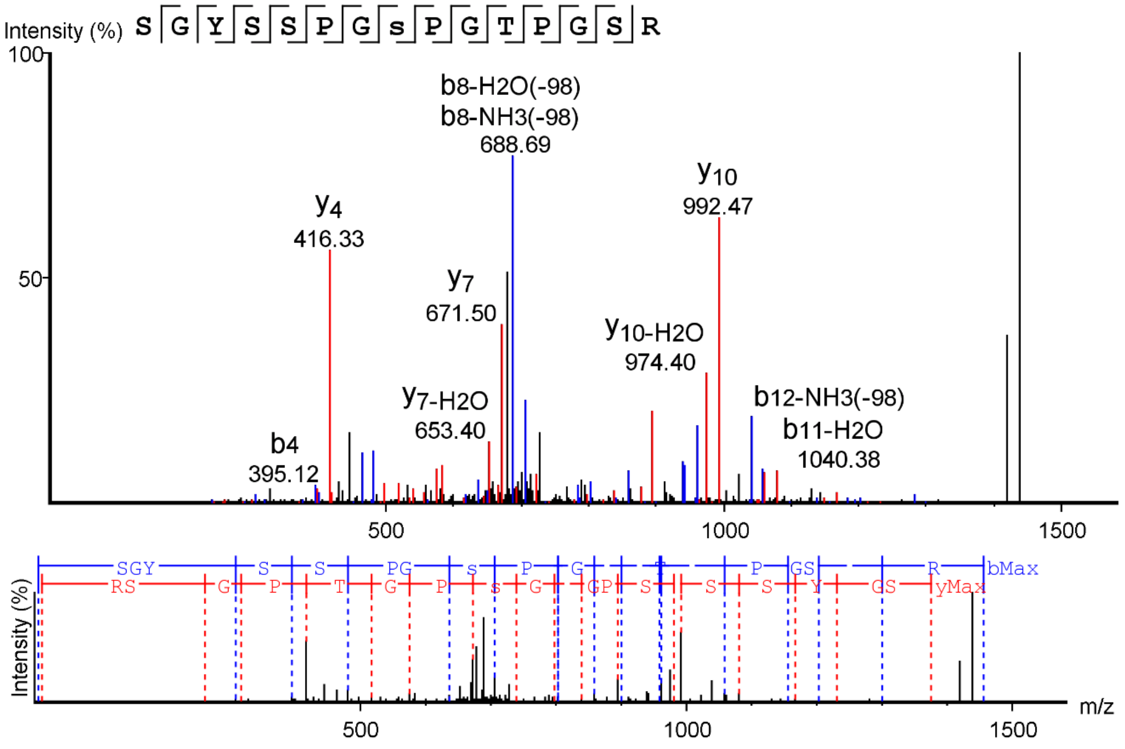

Ion table and mass error map

#	b	b-H2O	b-NH3	b (2+)	Seq	y	y-H2O	y-NH3	y (2+)	#
1	88.04	70.03	71.01	44.52	S					15
2	145.06	127.05	128.03	73.03	G	1386.57	1368.56	1369.54	693.74	14
3	308.07	290.23	291.10	154.56	Y	1329.55	1311.54	1312.52	665.70	13
4	395.12	377.24	378.13	198.08	S	1166.55	1148.45	1149.27	584.01	12
5	482.22	464.27	465.16	242.12	S	1079.52	1061.42	1062.42	540.45	11
6	579.24	561.23	561.98	290.23	P	992.47	974.40	975.39	496.86	10
7	636.25	618.42	619.24	319.07	G	895.37	877.49	878.34	448.18	9
8	803.29	784.90	786.58	402.13	S(+79.97)	838.45	820.53	821.60	419.30	8
9	899.93	882.30	883.08	451.18	P	671.50	653.40	654.32	336.17	7
10	957.42	939.15	939.97	478.67	G	574.75	556.40	557.27	287.35	6
11	1058.35	1040.38	1041.36	529.69	T	517.49	499.26	500.25	259.30	5
12	1155.53	1137.19	1137.82	578.22	P	416.33	398.24	399.20	208.61	4
13	1212.46	1194.57	1195.23	606.73	G	319.07	301.11	302.32	160.09	3
14	1299.60	1281.53	1282.46	650.20	S	262.07	244.11	245.36	131.58	2
15					R	175.12	157.11	158.09	88.06	1

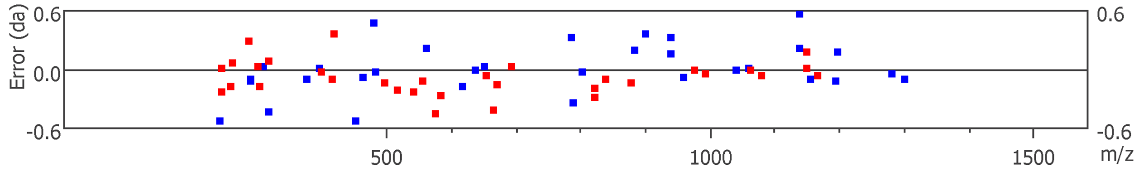

S7.B: peptide TPPAPKT(+79.97)PPSSGEPPK, mass 1666.7966

Annotated spectrum with alignment

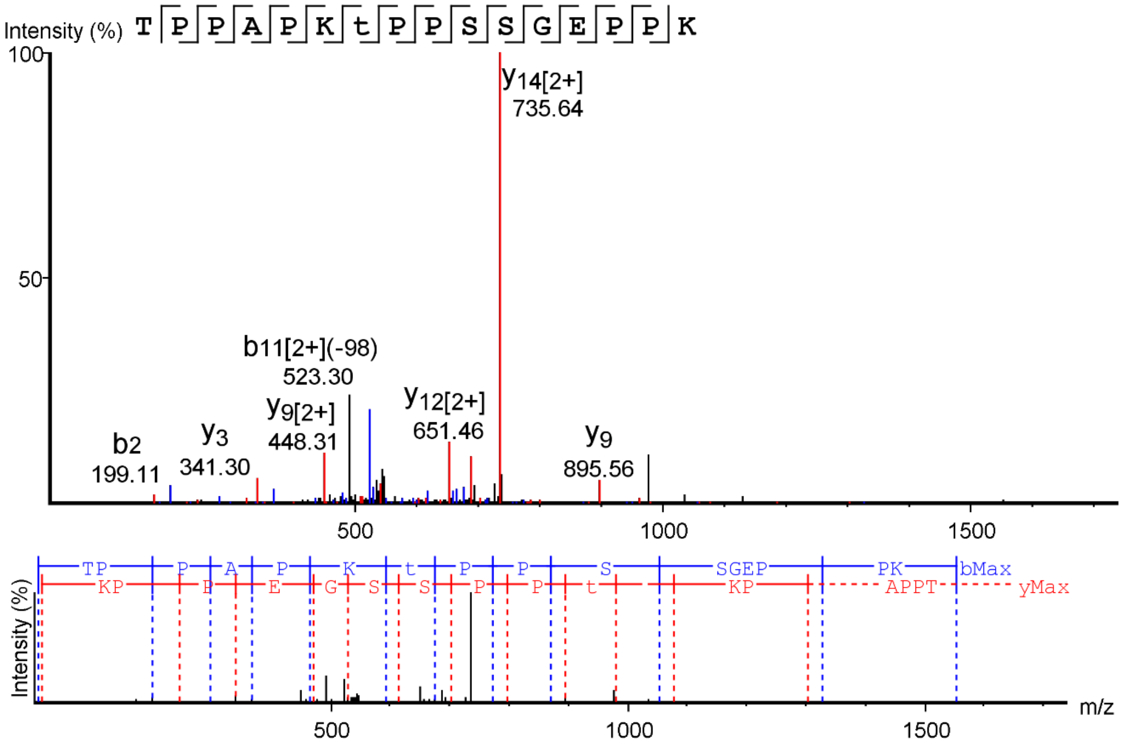

Ion table and mass error map

#	b	b-H2O	b-NH3	b (2+)	Seq	y	y-H2O	y-NH3	y (2+)	#
1	102.06	84.04	85.03	51.53	T					16
2	199.11	181.13	182.08	100.05	P	1566.76	1548.75	1549.73	783.43	15
3	296.24	278.32	279.13	148.58	P	1469.70	1451.69	1452.68	735.64	14
4	367.26	349.04	350.17	184.10	A	1372.65	1354.64	1355.62	686.77	13
5	463.90	446.24	447.22	232.22	P	1301.92	1283.60	1284.59	651.46	12
6	592.48	574.32	575.32	296.24	K	1204.56	1186.55	1187.53	602.51	11
7	773.54	755.46	756.33	387.18	T(+79.97)	1076.53	1058.60	1059.83	539.10	10
8	870.77	852.40	853.39	435.52	P	895.56	877.59	878.35	448.31	9
9	967.47	949.45	950.36	484.63	P	798.41	779.86	781.51	399.95	8
10	1054.55	1036.49	1037.47	527.56	S	701.62	683.34	684.32	351.46	7
11	1141.53	1123.52	1124.50	571.28	S	614.49	596.30	597.56	307.66	6
12	1198.55	1180.54	1181.52	600.17	G	527.56	508.93	509.91	264.14	5
13	1327.59	1309.58	1310.57	664.53	E	470.79	452.49	453.23	235.63	4
14	1424.65	1406.64	1407.62	713.09	P	341.30	323.32	324.19	171.12	3
15	1521.70	1503.69	1504.67	761.78	P	244.21	226.25	227.14	122.58	2
16					K	147.11	129.10	130.09	74.06	1

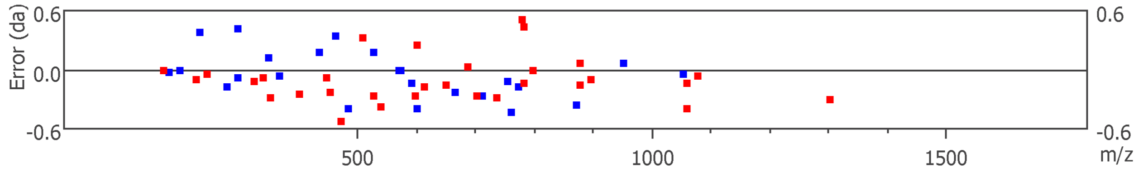

S7.C: peptide KVAVVRT(+79.97)PPKSPSSAK, mass 1730.9443

Annotated spectrum with alignment

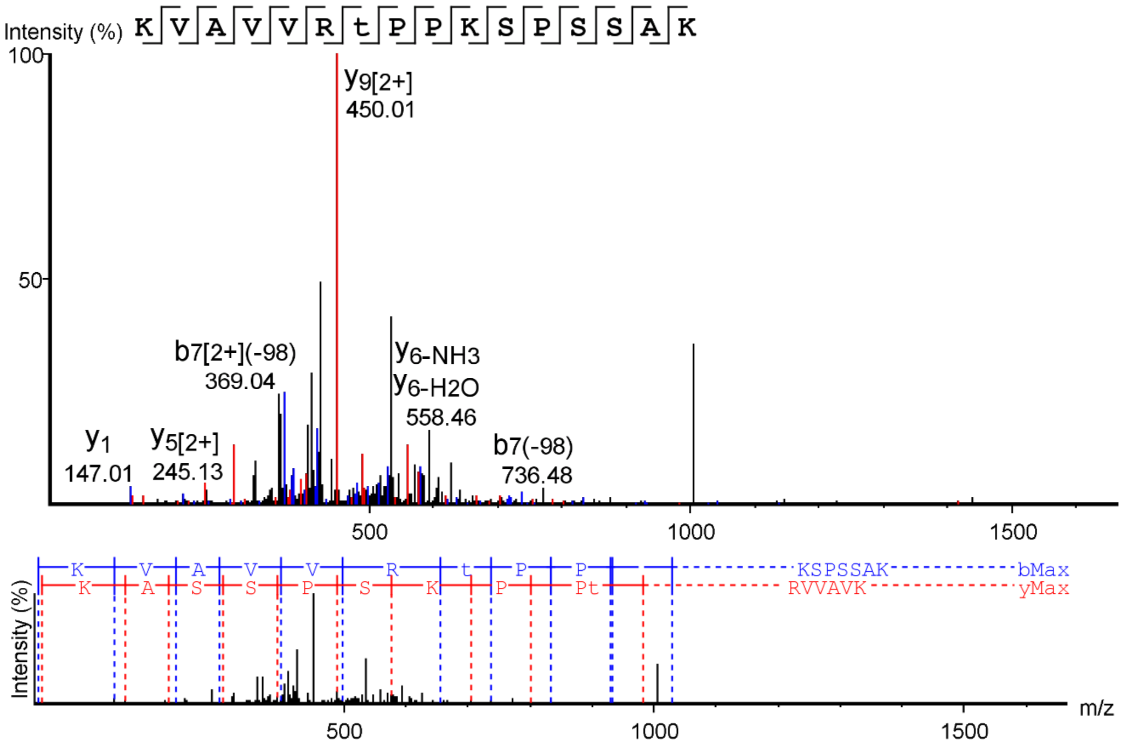

Ion table and mass error map

#	b	b-H2O	b-NH3	b (2+)	Seq	y	y-H2O	y-NH3	y (2+)	#
1	129.14	111.09	112.08	65.05	K					16
2	228.25	210.01	211.14	114.59	V	1603.86	1585.85	1586.83	802.32	15
3	299.36	281.20	282.27	150.10	A	1504.79	1486.78	1487.76	753.21	14
4	398.41	379.89	380.97	199.64	V	1433.75	1415.99	1416.72	717.89	13
5	497.29	478.75	480.34	249.19	V	1334.68	1316.67	1317.66	668.00	12
6	653.86	635.53	636.42	327.24	R	1235.61	1217.60	1218.59	618.65	11
7	834.51	816.45	817.65	418.11	T(+79.97)	1079.51	1061.50	1062.49	540.71	10
8	931.78	913.50	914.68	466.47	P	898.50	880.49	881.47	450.01	9
9	1028.77	1010.56	1011.54	514.92	P	801.59	783.44	785.01	401.55	8
10	1156.66	1138.51	1139.63	578.77	K	704.39	686.91	686.91	352.48	7
11	1243.69	1225.68	1226.67	622.62	S	576.45	558.46	559.75	288.87	6
12	1340.75	1322.73	1323.72	671.17	P	489.32	471.45	472.38	245.13	5
13	1427.78	1409.77	1410.75	714.43	S	392.22	374.29	375.44	196.61	4
14	1514.81	1496.80	1497.78	758.35	S	305.36	287.25	288.15	153.09	3
15	1585.85	1567.84	1568.82	793.42	A	218.16	200.44	201.12	109.57	2
16					K	147.01	129.14	130.05	74.06	1

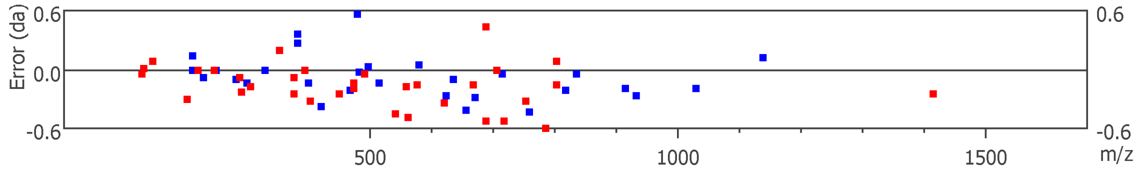

S7.D: peptide TPPAPKT(+79.97)PPSSGEPPKSGDR, mass 2081.9783

Annotated spectrum with alignment

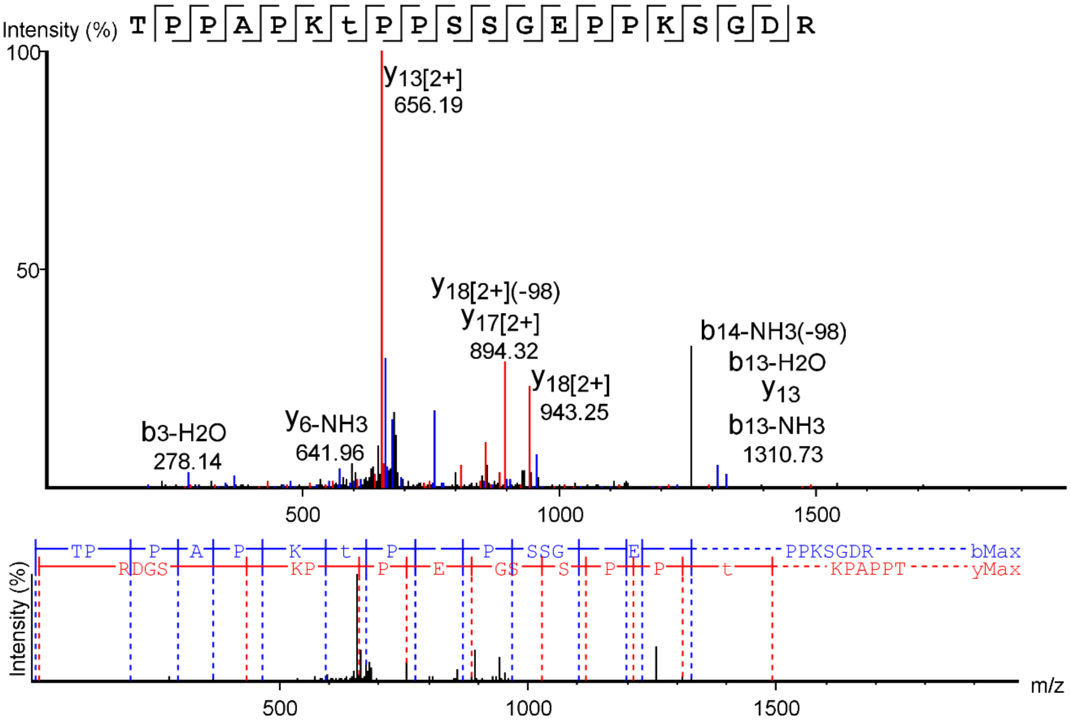

Ion table and mass error map

#	b	b-H2O	b-NH3	b (2+)	Seq	y	y-H2O	y-NH3	y (2+)	#
1	102.06	84.04	85.03	51.53	T					20
2	199.08	181.10	182.08	100.05	P	1981.94	1963.93	1964.91	991.67	19
3	296.38	278.14	279.13	148.58	P	1884.89	1866.87	1867.86	943.25	18
4	367.16	349.32	350.17	184.10	A	1787.83	1769.82	1770.81	894.32	17
5	464.54	446.78	446.78	232.63	P	1716.80	1698.78	1699.77	858.92	16
6	592.28	574.47	575.32	296.38	K	1619.74	1601.73	1602.72	810.09	15
7	773.32	755.61	756.63	387.18	T(+79.97)	1492.07	1473.75	1474.62	746.67	14
8	870.31	852.40	853.77	435.71	P	1310.73	1292.74	1293.60	656.19	13
9	967.06	949.25	950.44	483.69	P	1213.63	1195.66	1196.55	607.63	12
10	1054.50	1036.49	1037.47	528.34	S	1116.31	1098.52	1099.68	558.96	11
11	1141.53	1123.52	1124.50	570.99	S	1029.33	1011.27	1012.50	515.59	10
12	1198.63	1180.54	1181.82	599.79	G	942.46	924.99	924.99	471.29	9
13	1327.82	1309.52	1310.73	664.59	E	885.74	867.49	868.42	443.22	8
14	1424.65	1406.64	1407.62	712.82	P	756.63	738.55	739.67	378.70	7
15	1521.70	1503.69	1504.67	761.35	P	659.58	641.34	641.96	330.77	6
16	1649.79	1631.78	1632.77	825.40	K	562.29	544.75	544.75	281.38	5
17	1736.83	1718.82	1719.80	868.91	S	434.13	416.19	417.61	217.60	4
18	1793.85	1775.84	1776.82	897.27	G	347.17	329.11	330.14	174.08	3
19	1908.87	1890.86	1891.85	955.29	D	290.15	272.14	273.20	145.57	2
20					R	175.12	157.11	158.09	88.06	1

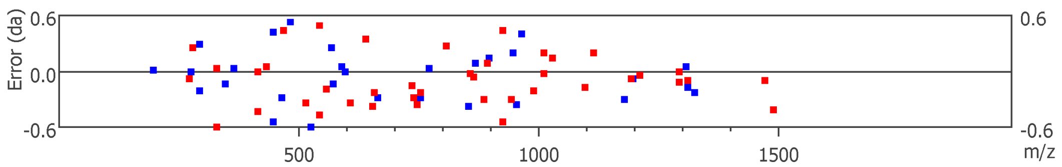

Supplement: Supplementary file 1 [file mmc1.pdf]
